# Metal-induced changes in the early-life gut microbial composition and life-history traits in three insectivorous passerines

**DOI:** 10.1007/s11356-025-37181-x

**Published:** 2025-11-21

**Authors:** Miia J. Rainio, Lyydia Leino, Eero Vesterinen, Pablo Sánchez Virosta, Pere Puigbò, Tapio Eeva

**Affiliations:** 1 Department of Biology, University of Turku, 20014 Turku, Finland; 2 Department of Biology, Norwegian University of Science and Technology, Trondheim, Norway; 3 Department of Biochemistry and Biotechnology, Rovira I Virgili University, Tarragona, Catalonia Spain; 4 Eurecat, Technology Centre of Catalonia, Reus, Catalonia Spain

**Keywords:** Blue tit, Microbial diversity, Great tit, Gut bacteria, Pollution, Pied flycatcher

## Abstract

Pollution-induced changes in gut microbiota may have serious effects on avian health and physiology. Particularly, early-life exposure to environmental pollutants may influence the gut microbiota development and microbiota-dependent physiological functions. We studied the associations of metal pollution with early-life microbial environment of wild passerines by exploring gut bacterial diversity and community composition in nestlings by 16S rRNA sequencing. Samples were collected from three common model species, great tits (*Parus major*), blue tits (*Cyanistes caeruleus*), and pied flycatchers (*Ficedula hypoleuca*) breeding near a copper-nickel smelter, an area with the highest rates of metal pollution in Finland. Responses of microbiota to pollution and microclimate were measured and connected to ecological measures (brood size, growth, fledging success) of nestling performance. The most abundant bacterial phyla across species were Firmicutes, Proteobacteria and Actinobacteria. Our results showed that the faecal microbial alpha diversity decreased with increasing brood size in great tits and blue tits, while in pied flycatchers the diversity increased with increasing nestling growth. However, alpha diversity did not differ between the polluted and control areas. The faecal microbial communities (beta diversity) differed between the species and study areas, and several bacterial orders were associated with metal concentrations, temperature, body mass, or fledging success. Differential abundance analyses showed species-specific differences in microbial abundances between polluted and control areas, e.g., *Catellicoccus* and *Serratia* showing elevated abundance within the polluted area. The results suggest that metal pollution may alter the early-life bacterial communities in small passerine species living in metal-polluted areas.

**Supplementary Information:**

The online version contains supplementary material available at 10.1007/s11356-025-37181-x.

## Introduction

Microbes play a significant role in terrestrial and aquatic ecosystems by acting as plant, animal, and human pathogens, but several microbes have beneficial and protective functions as well (Kohl 2012; Moreno et al. 2003). The balance in microbial communities is important, since they can help us restore natural ecosystems and maintain the well-being of their hosts (Angulo et al. 2019). Significance of microbiota to vertebrate health and fitness is an emerging topic in ecology, but the research has mostly focused on mammals. So far, relatively little is known about the factors shaping the establishment and dynamics of microbial communities in wild bird species and the role of environmental pollutants in the development of early-life microbial flora. The colonization of nestlings by environmental microbes begins soon after hatching (Lucas and Heeb 2005) via ingestion of adult saliva, food items or nest materials (Berger et al. 2003; Diez-Méndez et al. 2023; Kyle and Kyle 1993; Liukkonen et al. 2024; Mills et al. 1999; Somers et al. 2023), potentially affecting the balance between beneficial and harmful bacteria in host species.

Anthropogenic pollution is one potential factor affecting the microbial communities in animals, causing various health disorders and dysregulation of the immune system of animals and affecting negatively to the function of gut microbiota (Arun et al. 2021; Claus et al. 2016; Duan et al. 2020; Li et al. 2019). Although the interaction between environmental pollution and gut microbiota remains poorly understood, several contaminants are known to modify microbial composition and metabolism, potentially influencing host physiology and health (Claus et al. 2016). Bacteria-mediated metabolism of toxic compounds may also modulate their toxicity, and disruption of the normal microbial detoxification processes could impair stress responses, health, and fitness in wild animals.

Metals are among the most widespread anthropogenic pollutants and well known for their antimicrobial activity (Lemire et al. 2013). Metal toxicity affects microbial abundance, diversity, and activity (Ayangbenro and Babalola 2017; Zhang et al. 2023), although certain metals—such as iron, cobalt, nickel, copper, and zinc—are essential for most organisms (Frei et al. 2023). Non-absorbed metals may persist in the gut microenvironment, directly influencing the microbiota and the physiology of developing individuals (Breton et al. 2013). Understanding the mechanisms through which such metals affect host–microbe interactions is therefore crucial for assessing their ecological and health impacts.

The gut microbiota, comprising of bacteria, archaea, fungi and viruses and provides crucial physiological functions that host organisms cannot develop by themselves (Kohl 2012). Coevolving as a mutualistic partner, the microbiota supports host metabolism, immunity, and development, while imbalances may contribute to metabolic disorders under environmental stressors (Breton et al. 2013; Kogut 2013; Kohl 2012). Diet is one of the key factors modulating the gut microbiota composition within and among species (Bodawatta et al. 2022b; Glunder 2002). For example, insectivorous species often feed their nestlings with caterpillars, yet variation in prey species and contaminant accumulation across habitats can influence host bacterial communities (Goodenough and Stallwood 2010). Gut microbes also correlate with nestling phenotype and condition, including body size, wing asymmetry, and overall health (Mills et al. 1999; Moreno et al. 2003), underscoring their importance in avian development.

Avian gut microbiota is typically dominated by the phyla Proteobacteria, Firmicutes and Actinobacteria (Bodawatta et al. 2022a; Grond et al. 2018; Kropáčková et al. 2017; Liukkonen et al. 2023), and the most common cloacal bacteria genera include *Enterococcus*, *Escherichia,* and *Enterobacter*. *Enterococci*, for example, are part of the normal bacteria flora, acting as either opportunistic pathogens or beneficial growth promoters and components of probiotics (Moreno et al. 2003). Although avian microbiota possesses broad enzymatic capacity to metabolize environmental contaminants, pollutants may still disrupt key microbial functions, posing particular risks during early life stages when nestlings are most vulnerable.

In this study, we examine the association of metal pollution on early-life gut microbiota in wild passerine birds and whether microbiota changes are linked to nestling performance. Previous studies have highlighted the importance of indirect effects of pollution on avian physiology and fitness (Koivula et al. 2011), yet metal-induced alterations in gut microbiota remain poorly understood (Zhang et al. 2023; Liu et al. 2025). Our findings provide new insights into the reduced nestling growth and lower breeding success often observed in urban and industrial bird populations (Chamberlain et al. 2009), even in the absence of clear direct toxic effects on physiology and performance.

We address the following questions: (1) Does metal pollution affect the species microbial diversity (alpha diversity) and community composition (beta diversity) of nestling gut microbiota? Given that metal exposure can alter microbial diversity and metabolism (Liu et al. 2021; Zhang et al. 2023), we expect differences in gut microbiota between polluted and control areas. (2) Do species differ in their gut microbiota in response to metal pollution? As our three study species differ in phenology, diet, nest composition, and tolerance to pollution, we predict species-specific differences in microbiota composition and responses. (3) Do individual bacterial taxa vary in abundance with faecal metal concentrations? Some bacteria can tolerate or resist certain metals, thus potentially varying in abundance between the polluted and control areas.

## Materials and methods

### Study area and study species

The fieldwork was conducted in an established nest box study area close to a copper-nickel smelter in Harjavalta (61°20′ N, 22°10′ E), an area with the highest rates of metal pollution in Finland. A long-term nest box scheme has been running in this area since 1991 and there is ample information on metal exposure levels (e.g., in bird faeces, Berglund et al. 2015), genetic and physiological effects (Koivula et al. 2011), and fitness effects (Eeva and Lehikoinen 1996) on birds from this area. Nine study sites, each with 20–60 nest boxes, were divided to polluted (4 sites < 2 km from the smelter) and control (5 sites > 5 km from the smelter) areas, based on the previous data showing that the metal concentrations decrease exponentially with increasing distance to the pollution source (Koivula et al. 2011). The habitat type in all study sites was relatively barren forest dominated by Scots pine (*Pinus sylvestris*), thus minimizing habitat-related variation between the areas. However, vegetation near the smelter has suffered from the long-term pollution and the ground layer vegetation cover is patchy at more heavily polluted locations (Kiikkilä 2003). All nest boxes were carefully cleaned from old nest materials before the breeding season.

Three insectivorous cavity-breeding passerine birds, the great tit (*Parus major*), blue tit (*Cyanistes caeruleus*), and pied flycatcher (*Ficedula hypoleuca*) were used as model species. Great tit and blue tit are resident species in Finland, while pied flycatchers winter in Western Africa (Lundberg and Alatalo 1992). Great tit and blue tit share more similar breeding ecology, typically starting egg laying in mid-May, whereas pied flycatchers begin laying in late May. All three species rely primarily on caterpillars and other insects to feed their nestlings, with pied flycatcher additionally providing flying insects and spiders. Differences in phenology, habitat preferences, and reproductive strategies result in distinct ecological responses to environmental variation, such as changes in food availability. The two tit species use similar nest materials, mainly moss and animal hair, while pied flycatcher primarily use tree bark and dry grasses (Lundberg and Alatalo 1992; Perrins 1979; Visser et al. 2006). All three species are abundant in our study areas and breed in the nest boxes, making them ideal species to study the associations between anthropogenic pollution and early-life microbiota of the birds.

### Sampling in the field

During the breeding season 2021, we randomly selected 44 nests from polluted sites (great tit *n* = 16, blue tit *n* = 13 and pied flycatcher *n* = 15) and 45 nests from control sites (great tit *n* = 15, blue tit *n* = 15 and pied flycatcher *n* = 15) for sampling. The timing of breeding was standardized by avoiding late or replacement nests. Small temperature and humidity data loggers (iButtons® DS1923-F5# Hygrochron, Maxim Integrated Products) were placed inside the nest boxes to measure ambient growing conditions for microbes every three hours. The nest boxes were inspected from the beginning of the breeding season until the fledging phase to collect data on hatchability, brood size, growth and fledgling success. Any microbial cross-contamination was avoided by wearing sterile gloves when there was a need to touch the nest or nestlings. At the average age of 8 days (range 5–11 days), the chicks were individually ringed with aluminium rings and the body mass (Pesola spring balance, g) and wing length (mm) were measured. In addition, a pooled faecal sample was collected from each brood for gut microbiota sequencing and metal analyses by sampling each individual separately through gentle cloacal pressure to stimulate defecation when it did not occur spontaneously. Brood-level analyses account for a broader range of dietary inputs among chicks within a brood and may better represent the overall influence of diet on the faecal microbiota. Fresh faecal samples were stored in sterile polypropylene tubes, placed in a portable cooler in the field and frozen at − 20 °C until the final storage at − 80 °C before the DNA extractions. The experiments were conducted under licenses of the Regional State Administrative Agency for Southern Finland (license number ESAVI/3021/04.10.07/2017) and the Centre for Economic Development, Transport and the Environment of Southwest Finland (licence number VARELY/3622/2017).

### Metal analyses

The concentrations of five metals (Arsenic As, Cadmium Cd, Copper Cu, Nickel Ni and Zinc Zn) were determined from the faecal samples in the CEBAS-CSIC laboratory (University of Murcia, Spain). Metal concentrations were analysed with an inductively coupled plasma optical emission spectrometer (ICP-OES, Thermo ICAP 6500 Duo) with the quantification limit of 0.01 ppm. Faecal samples (0.1–0.2 g, dry weight) were placed in digestion tubes to which a mixture of 4 ml HNO_3_ (70%) and 1 ml H_2_O_2_ (33%) was added. The sample was then submitted to a progressive thermal treatment and, after a microwave procedure, the sample was diluted in ultrapure water before the analysis (see Espín et al. 2016). Precision of the method was tested with certified reference material (TORT-2, lobster hepatopancreas, National Research Council Canada) and element recoveries were found to vary between 115 and 169%. Because of the relatively high recovery values, we did not use absolute values in the analyses, but instead principal components calculated from those five metals as an index of metal exposure levels.

### DNA extraction and library preparation

All molecular work including DNA extraction, NGS library preparation, and sequencing as well as bioinformatics were carried out as turnkey service by DNA analysis company Bioname (Turku, Finland). In summary, bacterial DNA was extracted from the faecal samples using Quick-DNA Fecal/Soil microbe Miniprep Kit (Zymo Research). For each original faecal DNA extract, we carried out two independent PCRs. Both of these replicates were processed the same way, and indexed separately. The libraries were prepared through two subsequent PCR stages. In the first locus-specific stage, the microbial 16S ribosomal RNA gene V4 region was amplified using one primer pair, designed to amplify a highly variable short gene region to enable bacterial taxa identification. We used forward primer 515FB (GTGYCAGCMGCCGCGGTAA, see Parada et al. 2016; Walters et al. 2016) and reverse primer 806RB (GGACTACNVGGGTWTCTAAT, see Apprill et al. 2015; Caporaso et al. 2011). To increase the amplicon library diversity, each primer was used as four different versions, so that they included 0–4 so called heterogeneity spacer nucleotides between the linker-tag and the actual locus-specific oligo. In the second library-PCR stage, 1 st PCR product was used as a template for the second PCR, in which we used a dual indexing strategy, where each reaction (including technical replicates) was prepared with a unique combination of forward and reverse indices. All index sets were balanced perfectly so that each nucleotide position included a signal for each channel in the sequencing. The sequencing was performed on the Illumina NovaSeq6000 SP Flowcell using 2 × 250 bp paired-end read length (Illumina Inc. San Diego, California, USA) in the Finnish Functional Genomics Centre (FFGC, University of Turku, Finland). Detailed molecular workflow is available as Supplement text 1: “Molecular analysis”.

### Bioinformatics

Our bioinformatics workflow closely followed Kaunisto et al. (2020). Shortly, the raw reads were trimmed, merged, the PCR primers were removed using the software CUTADAPT 2.7 (Martin 2011), reads were dereplicated, and then collapsed into sequence variants (ZOTUs) using ‘unoise3’ algorithm (default options: minsize = 8 and alpha = 2) in USEARCH 11 (Edgar 2010). The similarity threshold is not set when calling for sequence variants, such as ZOTUs or ASVs. In practice, the ZOTUs are Zero-radius OTUs, and the sequence similarity is 100%. The unoise3 algorithm performs error-correction (denoising) on amplicon reads, and removes chimeras. The abundance information of the unique reads is used to deduct the correct amplicon from the spurious ones.

The number of ZOTUs in each sample was assessed and all ZOTUs were assigned to taxa using USEARCH/VSEARCH SINTAX algorithm using pre-built database (*16S RDP training set v16*) downloaded from https://drive5.com/usearch/manual/sintax_downloads.html; Edgar and Flyvbjerg, 2015). Finally, reads were filtered for non-target reads, such as Chloroplast DNA. Low abundance ZOTU’s (= read count less than 2) were removed from the data. After filtering, majority of the reads were retained in the data, including 19,208,556 reads. Prior to rarefaction and final filtering, we had an average 220,788 reads per sample and the read counts varied a lot between the samples (min = 935 reads; max = 581,108 reads per sample); only one sample had fewer than 1000 reads. Detailed bioinformatics have been collected in the Supplement text 2: “Bioinformatics”.

### Statistics

Most of the statistical analyses were performed either with R (v. 4.2.3; R Core Team 2023) or SAS 9.4 statistical software (SAS 2013). Prior to further statistical analyses, the data was transformed to a phyloseq object for R. Subsequently, the microbial data was rarefied to a depth of 29,000 reads (phyloseq 1.42.0 in R, (McMurdie and Holmes 2013) to account for the differences in the sequencing depth. The rarefaction cut off was determined based on the plateauing of rarefaction curves, i.e., the point where no significant number of additional taxa would have been found with a greater sequencing depth (Fig. S1). The final data included 6918 taxa and 84 samples in total.

Since the faecal metal concentrations correlated with each other, we calculated principal components (PC) from the metal data (Ni, Cu, As, Cd and Zn) with SAS 9.4. The first principal component (PC1_M_; Ni, Cu, As) explained 56.2% (eigenvalue 2.81) of the variation in our metal data and was used in the models as an explanatory variable to describe the general level of metal exposure. The second component (PC2_M_; Zn, Cd) explained 23.4% (eigenvalue 1.17) of the variation in our data. Promax rotation method, which allows components to be correlated, was used in the analysis.

#### Differential abundance analyses

Based on literature, five commonly used differential abundance (DA) estimators ALDEx2 (1.30.0) (Fernandes et al. 2013), ANCOM-BC2 (2.0.2) (Lin and Das Peddada 2020), Corncob (0.3.1) (Martin et al. 2020), DESeq2 (1.38.3) (Love et al. 2014) and LinDA (0.1.0) (Zhou et al. 2022) were used to identify the bacteria affected by study area (polluted, control) at different taxonomic levels: phylum, class, order, family and genus. Non-rarefied observed abundances were used as input and the estimators queried at a significance level of *p* < 0.05 after Benjamini–Hochberg adjustment. Prior to the DA analyses, the prevalence threshold was set to 10% by species. Nearing et al. (2022) have shown a high variation between the DA estimators across numerous 16S rRNA datasets, which is why we used the consensus of multiple pre-selected estimators for the analysis. A response was considered significant if at least three of the five estimators indicated significance. Used estimators calculate the results a different way, also using different data transformation techniques, which may affect the results. ALDEx2 and ANCOM-B2 (more conservative tests) use Wilcoxon rank-sum and Corncob and DESeq2 (more similar results with each other) use Wald test for hypothesis testing, while LinDA computes the *p*-values based on the bias-corrected regression coefficients (Nearing et al. 2022; Zhou et al. 2022). Considering our data, different DA estimators showed various amounts of differentially abundant taxa between the study areas. While DESeq2 and Corncob found 151 (63.6% unique to this estimator) and 128 (64.1% unique to this estimator) DA taxa in total (including all bird species and taxa), respectively, ANCOM-BC2 and LinDA found only 27 (18.5% unique to this estimator) and 25 (12.0% unique to this estimator) DA taxa, respectively. ALDEx2 found no differentially abundant taxa in any of the species.

#### Alpha diversity

Shannon diversity index (i.e., a number of bacterial ZOTUs and their abundance evenness within a sample), Chao1 richness (estimate of the total number of different bacterial ZOTUs in a sample) and observed richness (observed number of ZOTUs) were used as measures of alpha diversity of the nestling gut microbiota (microbiome 1.20.0. package in R, (Lahti and Shetty 2022). Thereafter, each diversity index was used as a response variable separately in the general (LM) or generalized (GLM) linear model (GLIMMIX procedure in SAS 9.4) using study area, temperature, nestling age and brood size at the time of sampling, and nestling relative body mass (RBM) as explanatory variables. The three species were analysed separately, since they differ in their feeding habits and migratory behaviour from each other. The RBM is a proportional (%) deviation of brood mean body mass from predicted mass based on long-term (years 1991–2022) growth curve from the same area. Using RBMs is reasonable for taking account of the slight variation in among-brood measurement ages. Temperature denotes average daily temperature inside the nest box from hatching to the brood age of 8 days, which was the average day for the microbial sampling. For Shannon index, we used beta distribution and logit link function, whereas for Chao1 index and observed richness, we used lognormal distribution with identity link function. Non-significant terms were dropped sequentially from the final model, but the study area (polluted, control) was always kept in the model, as this was our main study factor.

#### Beta diversity

Beta diversity was analysed with permutational multivariate analysis of variance (PERMANOVA with Bray–Curtis distances) using 999 permutations (“adonis2” in *vegan 2.6.4*, (Oksanen et al. 2022) to test the community dissimilarity between the study areas and species. In the model, we used study species, area, temperature (inside the nest box), age of nestlings at sampling, brood size, nestling body mass and study area × species interaction as explanatory factors. Pairwise comparisons between the species were calculated (*pairwise.adonis* 0.4.1, (Martinez Arbizu 2017) with Bray–Curtis method using 999 permutations and Bonferroni correction to adjust *p* values. To visualize the dissimilarity among species and study areas, we used principal coordinate analysis (PCoA) ordination based on Bray–Curtis distances (phyloseq 1.42.0, McMurdie and Holmes 2013). The samples were plotted according to the first and second component values. The analysis and visualization were performed for non-rarefied and rarefied data with relative abundance, but since the results did not change, we chose to use the rarefied data like in alpha diversity measurements.

#### Effects on life-history

We performed principal component analysis (PCA, SAS 9.4) from the log-transformed reads of 25 orders (thresholds of 5% prevalence and 1% abundance) to get fewer metrics of bacterial orders for further analyses and to visualize the bacterial data between the species and study areas. Based on the eigenvalues (> 1.5) and proportion (> 6%), only the first four components were chosen for the later analyses. The first component (PC1_B_; Acidimicrobiales^+^, Actinomycetales^+^, Gaiellales^+^, Solirubrobacterales^+^, Bacillales^+^, Planctomycetales^+^, Rhodobacterales^+^) explained 23.4% (eigenvalue 5.86), the second component (PC2_B_; Coriobacteriales^+^, Bacteroidales^+^, Clostridiales^+^, Erysipelotrichales^+^, Desulfovibrionales^+^, Sphingomonadales^**−**^) 12.5% (eigenvalue 3.12), the third component (PC3_B_; Caulobacterales^+^, Burgholderiales^+^) 7.4% (eigenvalue 1.86) and the fourth component (PC4_B_; Chlamydiales^+^, Legionellales^+^, Entomoplasmatales^+^) 6.6% (eigenvalue 1.64) of the variation in our data. The + and − signs refer to positive and negative loadings of the bacteria in each component. For visualization, we used a biplot based on the bacterial PCs (SAS). Those four components were further used in separate models as dependent variables to describe the gut bacterial composition of the nestlings.

To investigate the variation in gut bacteria abundances, we performed GLM separately for bacterial principal components PC1_B_ (GLM with beta distribution and logit link function), PC2_B_, PC3_B_ and PC4_B_ (LM with Gaussian distribution and identity link function) in SAS. We used PC1_M_ of the metals, species, brood size at sampling time, temperature (inside the nest box), sampling date, and RBM as explanatory variables in the models. Non-significant terms were dropped sequentially from the final model, but the PC1_M_ was always kept in the model, as this was our main study factor. Tukey’s test was used for post hoc pairwise comparisons. We assessed the collinearity of the main predictors by variance inflation factor (VIF) analyses.

We further tested the association of metals with fledging success (fledglings/hatchlings) (GLM with binomial distribution and logit link function) and RBM (LM with Gaussian distribution and identity link function). The models were performed separately for each study species, using PC1_M_ of metals, nestling body mass (fledging success model only), temperature inside the nest box, PC1_B_ and PC2_B_ of faecal microbes at order level as explanatory variables in the model.

Based on the DA results, the microbes that significantly differed between the study areas were chosen for the further analyses to see whether those microbes show potential association on fledging success (probability of a hatchling to fledge; GLM with binomial distribution and logit link function, SAS) and RBM (LM with Gaussian distribution and identity link function, SAS).

## Results

### Core gut microbiota

**Fig. 1:**
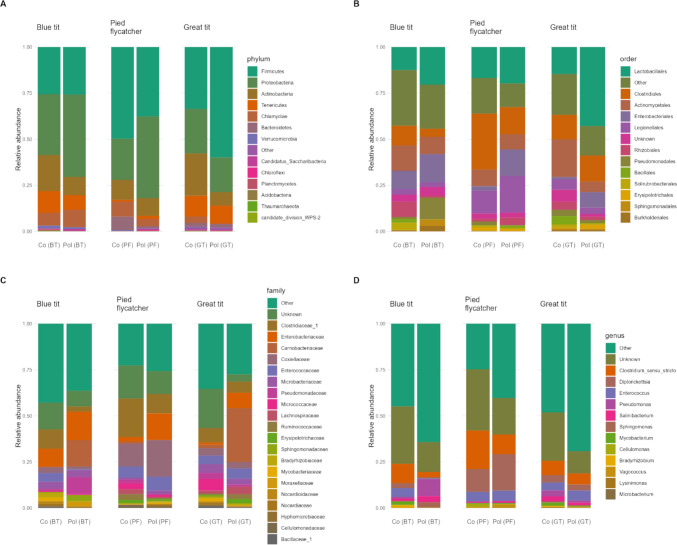
Mean relative abundance of the bacteria at different taxonomic levels (**A**) phylum, (**B**) order, (**C**) family and (**D**) genus in blue tit (*Cyanistes caeruleus*), great tit (*Parus major*) and pied flycatcher (*Ficedula hypoleuca*) nestlings in the polluted (Pol) and control (Co) areas. Analyses were performed with samples rarefied to read depth of 29,000. Taxa with the prevalence threshold of < 10% across all samples were assigned to “Other” at phylum level and < 50% at other taxonomic levels (order, family, genus)

Firmicutes (39.6%), Proteobacteria (30.4%) and Actinobacteria (13.5%) were the most abundant bacterial phyla across all species (great tit, blue tit and pied flycatcher) and study areas followed by Tenericutes (7.1%), Clamydiae (5.1%) and Bacteroidetes (2.2%) (Fig. 1, Fig. S2). The three most common phyla were, in varying order, the same for all species. The most common phyla in great tits and pied flycatchers were Firmicutes (great tit: 46.7%, pied flycatcher: 43.7%), Proteobacteria (great tit: 21.5%, pied flycatcher: 33.4%) and Actinobacteria (great tit: 15.1%, pied flycatcher: 10.2%), whereas Proteobacteria (37.9%) dominated in blue tits, followed by Firmicutes (25.6%) and Actinobacteria (15.5%, see Table S1). The predominant bacterial orders in great tits and blue tits were Lactobacillales (great tit:28.8%, blue tit:15.8%), followed by Clostridiales (13.7%) and Actinomycetales (13.1%) in great tits, and Enterobacteriales (12.1%) and Actinomycetales (12%) in blue tits. In pied flycatchers, the orders Clostridiales (22.5%), Lactobacillales (18.3%) and Legionellales (16.1%) dominated the faecal microbiota (see Table S1). The species showed some more variation of bacterial taxa at genus level; *Catellicoccus* (13.0%), *Ureaplasma* (8.1%) and *Clostridium *sensu stricto (6.6%) dominated the faecal gut bacteria in great tits, whereas *Ureaplasma* (8.2%), *Clostridium *sensu stricto (6.8%) and *Buchnera* (6.2%) were the most dominant genera in blue tits. The most abundant faecal gut bacteria in pied flycatchers were *Diplorickettsia* (16.1%), *Clostridium *sensu stricto (15.7%) and *Lactobacillus* (5.9%) (Table S1).

### Differential abundance analyses

**Fig. 2:**
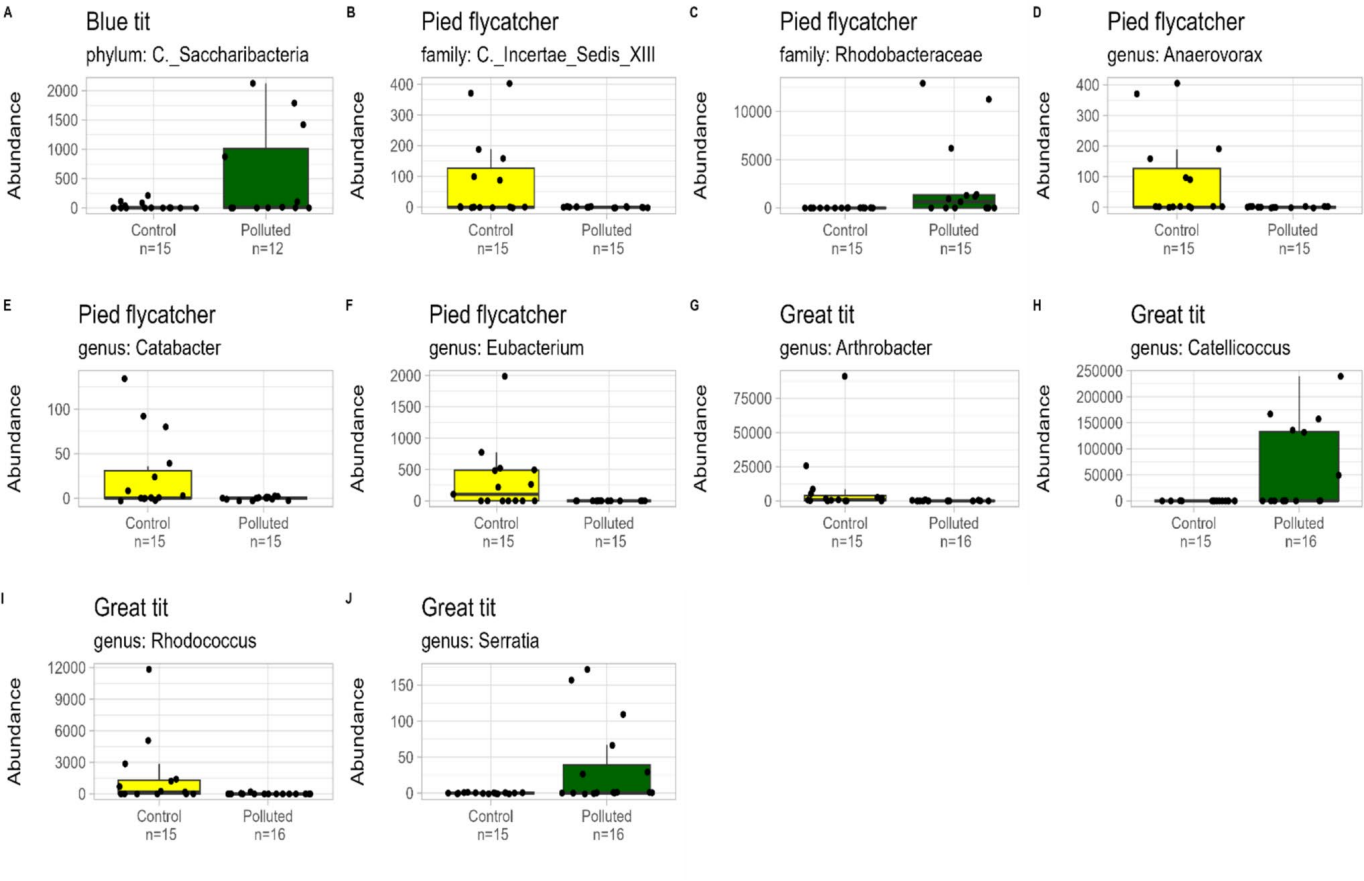
Abundance of the bacterial taxa at different levels (phylum, order, family and genus) showing differences between the polluted and control areas in blue tits, great tits and pied flycatchers (yellow = control area, green = polluted area). The differential abundance results are based on multiple estimators (ALDEx2, ANCOM-BC2, Corncob, DESeq2 and LinDA) on significance *p* < 0.05 after Benjamini–Hochberg adjustment

Five DA estimators were used to identify the bacteria associated with study area (polluted, control) at different taxonomic levels (Fig. 2). The results varied among the estimators, thus the responses of bacterial taxa in which three or more of the five estimators indicated a significant response were considered significant (see Table S2). In blue tits, the phylum Candidatus Saccharibacteria was more abundant in the polluted area compared to control area. In pied flycatchers, the order Rhodobacterales and family Rhodobacteraceae showed significantly greater abundance in polluted areas compared to control areas, whereas the family Clostridiales Incertae Sedis XIII was more abundant in control areas than in the polluted areas. At genus level, *Anaerovorax*, *Catabacter* and *Eubacterium* were more abundant in control areas compared to polluted areas in pied flycatchers, while in great tits *Arthrobacter* and *Rhodococcus* were more abundant, but *Catellicoccus* and *Serratia* less abundant in control areas compared to polluted areas. All significant comparisons (log2-fold change, SE, 95% CI and adj. *p*-value) are indicated in Table S2. The significant results of all DA estimators (log2-fold change, SE, 95% CI and adj. *p*-value) are shown as supplementary material (Table S3).

### Alpha diversity

**Fig. 3:**
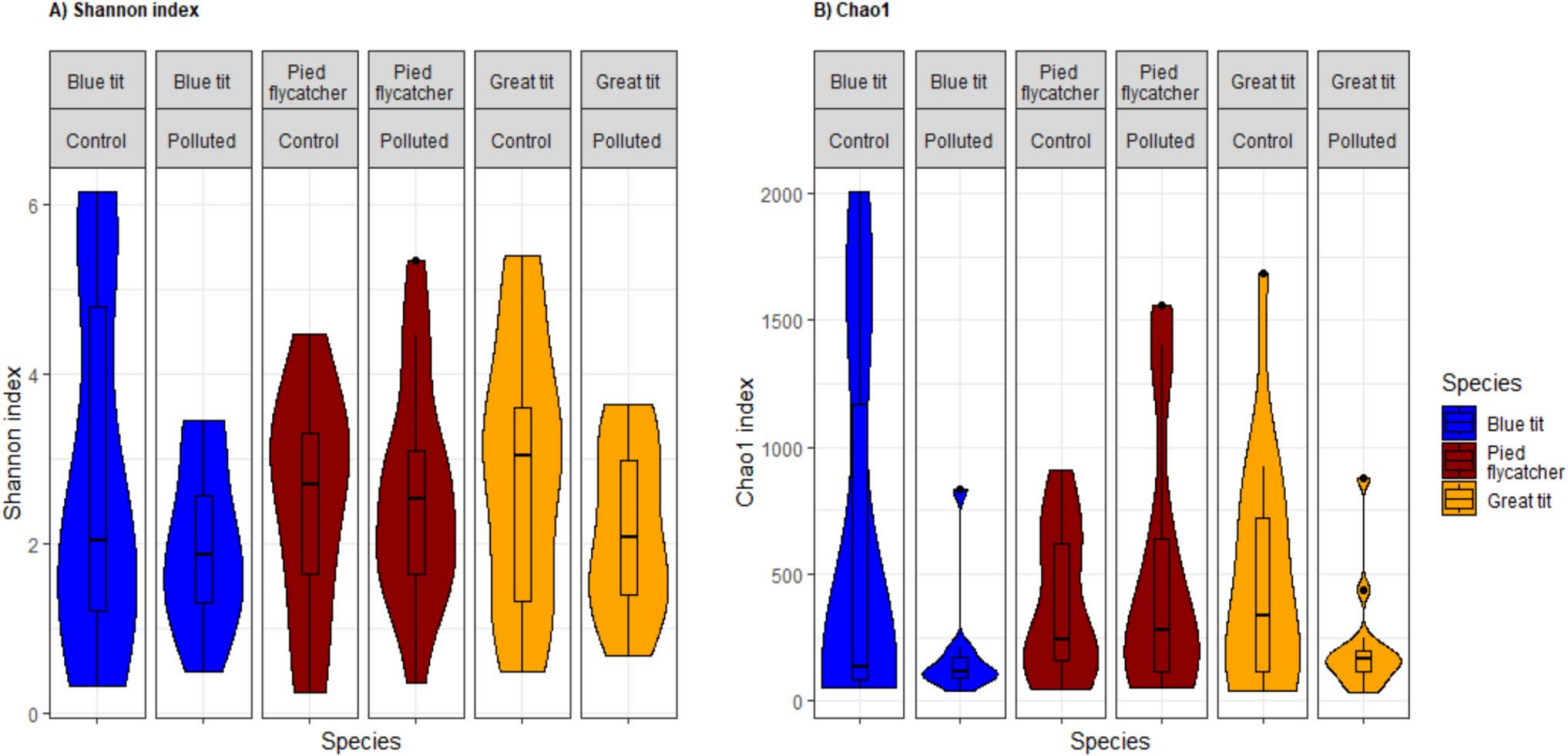
Mean (± SE) microbial alpha diversity based on (**A**) Shannon index and (**B**) estimated richness (Chao1) based on ZOTUs in blue tit (*Cyanistes caeruleus*), great tit (*Parus major*) and pied flycatcher (*Ficedula hypoleuca*) nestlings in polluted and control areas. The shape of the violins describes the kernel probability density at different diversity values. Analyses were performed on data rarefied to read depth of 29,000

We used Shannon index, Chao1 richness (Fig. 3) and observed richness as measures of alpha diversity of the nestling gut microbiota. Shannon diversity was negatively associated with brood size at sampling time in great tits and blue tits, and positively with temperature inside the nest box in blue tits, while no associations were found with RBM in the two tit species (Table 1). On the other hand, in pied flycatchers, higher RBM was associated with increased Shannon diversity, but not to brood size or temperature. Study area (polluted, control) or nestling age at sampling time were not associated with Shannon index in any of the species (Table 1). Observed richness and Chao1 richness index were not significantly associated with study area, nestling age, brood size at sampling time or temperature in any of the species. However, as opposed to tit species, in pied flycatchers, both richness indexes showed a significant positive association with RBM, suggesting higher overall microbial richness in well growing chicks.

**Table 1:** The association of area (polluted, control), temperature (inside the nest box), nestling age, brood size, relative body mass (RBM), and species and area*species interaction (PERMANOVA only) on alpha diversity (Shannon index, Chao1 richness and observed richness) and beta diversity (community composition) measures in the nestlings of great tits (*P. major*), blue tits (*C. caeruleus*) and pied flycatchers (*F. hypoleuca*). Non-significant terms were dropped sequentially from each model, starting from interaction. Terms left in the final model are shown in bold. Statistical significance was set as *p* < 0.05

Alpha-diversity (GLM)	Shannon index*		Chao1**		Obs. richness**	
Model	F_df_	p	F_df_	p	F_df_	p
			**Great tit**			
Area	**2.97**_**1, 27**_	**0.096**	**2.31**_**1, 28**_	**0.140**	**2.76**_**1, 28**_	**0.108**
Temperature	2.67_1, 26_	0.114	1.40_1, 25_	0.247	1.57_1, 24_	0.222
Age	0.19_1, 24_	0.669	1.19_1, 26_	0.286	1.28_1, 25_	0.268
Brood size	**4.52**_**1, 27**_ **est.** −0.11, **SE** 0.05	**0.043**	1.97_1, 27_	0.172	2.05_1, 27_	0.163
RBM	1.17_1, 25_	0.290	1.31_1, 24_	0.264	1.60_1, 26_	0.217
			**Blue tit**			
Area	**1.77**_**1, 20**_	**0.198**	**1.50**_**1, 22**_	**0.233**	**1.82**_**1, 22**_	**0.191**
Temperature	**5.52**_**1, 20**_ **est.** 0.14, **SE** 0.06	**0.029**	1.86_1, 21_	0.188	2.09_1, 21_	0.163
Age	1.80_1, 19_	0.196	0.53_1, 19_	0.475	0.86_1, 19_	0.366
Brood size	**5.42**_**1, 20**_ **est.** −0.18, **SE** 0.08	**0.031**	3.07_1, 20_	0.095	3.26_1, 20_	0.086
RBM	0.73_1, 18_	0.404	0.18_1, 18_	0.677	0.33_1, 18_	0.574
			**Pied flycatcher**			
Area	**0.45**_**1, 27**_	**0.508**	**0.35**_**1, 27**_	**0.560**	**0.26**_**1, 27**_	**0.612**
Temperature	0.55_1, 25_	0.467	0.01_1, 24_	0.939	0.00_1, 24_	0.997
Age	0.06_1, 24_	0.810	1.57_1, 26_	0.222	1.35_1, 26_	0.256
Brood size	1.11_1, 26_	0.302	0.04_1, 25_	0.843	0.03_1, 25_	0.854
RBM	**5.21**_**1, 27**_ **est.** 0.03, **SE** 0.02	**0.031**	**6.46**_**1, 27**_ **est.** 0.05, **SE** 0.02	**0.017**	**6.10**_**1, 27**_ **est.** 0.05, **SE** 0.02	**0.020**
**Beta-diversity**	PERMANOVA***					
**Model**	**F**_**df**_	**R**^**2**^	**p**			
Area	**2.32**_**1, 82**_	**0.025**	**0.001**			
Species	**2.14**_**2, 82**_	**0.047**	**0.001**			
Temperature	**1.69**_**1, 82**_	**0.018**	**0.002**			
Area*Species	1.05_2, 78_	0.023	0.321			
Age	1.28_1, 81_	0.014	0.087			
Brood size	1.23_1, 80_	0.013	0.117			
Body mass	**1.33**_**1, 82**_	**0.015**	**0.053**			

^*^GLM Beta distribution, logit link function

^**^GLM Lognormal distribution, identity link function

^***^PERMANOVA, reduced model (vegan, adonis2)

### Beta diversity

**Fig. 4:**
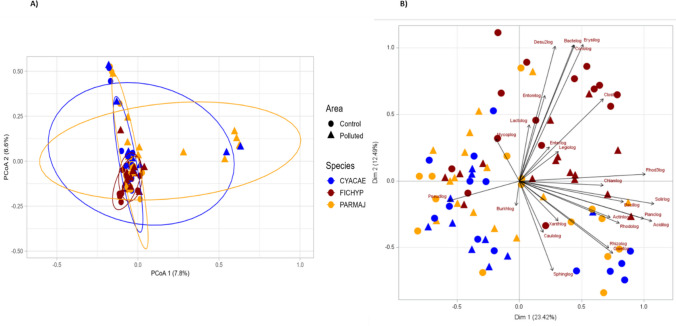
**A** Bacterial community composition changes between the species (blue tit, great tit and pied flycatcher) and study areas (polluted and control) shown as PCoA (Principal Coordinate Analysis) ordinations with Bray–Curtis dissimilarity index, and **B** Principal component biplot for bacterial orders in faecal samples of three bird species. The length and orientation of the vectors describe the impact of each order on individual PCs and their correlation with one another. In the PCoA plot each point represents a sample and closeness of the points indicates high similarity in the microbial community. The ellipses were drawn at 95% confidence interval. Analyses were performed with samples rarefied to read depth of 29,000. (CYACAE = blue tit*, n* = 23, PARMAJ = great tit*, n* = 30, FICHYP = pied flycatcher, *n* = 30)

Bray–Curtis dissimilarity-based microbial beta diversity was assessed to illustrate the differences in microbial communities between the study areas and study species (Fig. 4). The PCoA ordinations with Bray–Curtis dissimilarity index showed significant differences in microbial composition between the study areas (polluted, control) and study species (great tit, blue tit and pied flycatcher) and had significant association with temperature (PERMANOVA, Table 1). The pied flycatchers had a significantly different bacterial composition compared to great tits (F_df_ = 2.70_1_, p_adj_ = 0.003) and blue tits (F_df_ = 2.36_1_, p_adj_ = 0.003), while the two tit species did not differ significantly from each other (F_df_ = 1.18_1_, p_adj_ = 0.552). However, nestling age, brood size at sampling and nestling body mass had no association with the gut microbial composition (Table 1).

### Association of metals and life-history parameters on gut bacteria

Principal components (PC1_B_-PC4_B_) were calculated from the read counts of the bacterial orders and the first four components were further used in the models to study the association of faecal metal levels, temperature, and life-history parameters with bacterial taxa. The PC1_B_ (including orders Acidimicrobiales, Actinomycetales, Gaiellales, Solirubrobacterales, Bacillales, Planctomycetales, Rhodobacterales) increased significantly with faecal metal concentrations (PC1_M_ of metals), temperature, and RBM (used as a measure of growth, Table 2). Species did not differ from each other in relation to PC1_B_, and brood size and sampling date had no significant effect on PC1_B_ (Table 2). In contrast, PC2_B_ (including orders Coriobacteriales, Bacteroidales, Clostridiales, Erysipelotrichales, Desulfovibrionales, Sphingomonadales) had no significant association with faecal metal concentration but showed significant negative association with temperature and RBM (Table 2). On the other hand, PC2_B_ increased significantly with brood size and sampling date (Table 2). The PC2_B_ also differed significantly between the species; pied flycatcher showed higher bacterial abundance compared to great tits (Tukey’s test: t_df_ = 3.51_75_, p_adj_ = 0.002) and blue tits (Tukey’s test: t_df_ =  − 4.71_75_, p_adj_ =  < 0.0001), which did not differ significantly from each other (Tukey’s test: t_df_ =  − 1.78_75_, p_adj_ = 0.184). The PC3_B_, including Caulobacterales and Burkholderiales, did not show any association with faecal metal concentration or any other studied parameters (Table 2). The PC4_B_, which included bacterial orders Chlamydiales, Legionellales and Entomoplasmatales, was not affected by faecal metal concentrations, temperature, brood size or RBM, but showed a negative association with sampling date (Table 2). PC4_B_ differed significantly between the species, pied flycatchers having significantly higher bacterial abundance compared to great tits (t_df_ = 3.43_78_, p_adj_ = 0.003) and blue tits (t_df_ =  − 2.49_78_, p_adj_ = 0.039), while the two tit species did not differ significantly from each other (t_df_ = 0.95_78_, p_adj_ = 0.608).

**Table 2:** The association of metal level (PC1_M_), species (*P. major*, *C. caeruleus*, *F. hypoleuca*), brood size, temperature (inside the nest box), relative body mass (RBM) and sampling date on bacterial components (PC1_B_-PC4_B_) at order level. Non-significant terms were dropped sequentially from each model, starting from interaction. Terms left in the final model are shown in bold. Statistical significance was set as *p* < 0.05

Model	PC1_B_^*^		PC2_B_^**^		PC3_B_^**^		PC4_B_^**^	
	F_df_ (Est, SE)	p	F_df_ (Est, SE)	p	F_df_	p	F_df_ (Est, SE)	p
PC1_M_	**4.90**_**1, 79**_ **(est.** 0.06, **SE** 0.03**)**	**0.030**	**2.88**_**1, 75**_ **(est.** −0.32, **SE** 0.19**)**	**0.094**	**0.34**_**1, 81**_	**0.561**	**1.53**_**1, 78**_ **(est.** 0.17**, SE** 0.14**)**	**0.219**
Species	0.59_2, 76_	0.555	**11.38**_**2, 75**_	** < 0.0001**	2.69_2, 79_	0.074	**5.99**_**2, 78**_	**0.004**
Brood size	3.04_1, 78_	0.085	**5.58**_**1, 75**_ **(est.** 0.21, **SE** 0.09**)**	0.021	0.02_1, 76_	0.888	1.89_1, 77_	0.173
Temperature	**3.80**_**1, 79**_ **(est.** 0.02, **SE** 0.008**)**	**0.055**	**7.73**_**1, 75**_ **(est**. −0.24, **SE** 0.09**)**	**0.007**	0.97_1, 78_	0.327	0.09_1, 75_	0.770
RBM	**14.54**_**1, 79**_ **(est.** 0.008, **SE** 0.002**)**	**0.0003**	**4.77**_**1, 75**_ **(est.** −0.03, **SE** 0.01**)**	**0.032**	1.59_1, 77_	0.211	0.22_1, 76_	0.639
Sampling date	0.49_1, 75_	0.486	**7.71**_**1, 75**_ **(est.** 0.10, **SE** 0.04**)**	**0.007**	0.03_1, 75_	0.857	**7.24**_**1, 78**_ (**est.** −0.05, **SE** 0.02)	**0.009**

^*^ GLM (beta distribution, logit link function)

^**^LM (Gaussian distribution, Identity link function)

### Fledging success and RBM

Both fledging success and RBM were significantly decreased with increased faecal metal concentration (PC1_M_ of metals) in great tits and blue tits, but not in pied flycatchers (Table 3). In blue tits, the fledging success increased significantly with warmer temperature and was positively associated with PC2_B_ of bacterial taxa, while such effects were not found in the other species (Table 3). The fledging success had no association with nestling body mass or PC1_B_ in any of the three species (Table 3). In pied flycatchers, the RBM associated positively with PC1_B_, but negatively with PC2_B_, whereas such associations were not found in the tit species (Table 3)_._ The RBM was not significantly associated with temperature in any of the species (Table 3).

**Table 3:** The association of metal levels (PC1_M_) temperature (inside the nest box), body mass and bacterial components PC1_B_ and PC2_B_ on fledging success and relative body mass in the nestlings of great tits (*P. major*), blue tits (*C. caeruleus*) and pied flycatchers (*F. hypoleuca*). Non-significant terms were dropped sequentially from each model, starting from interaction. Terms left in the final model are shown in bold. Statistical significance was set as *p* < 0.05

	Great tit		Blue tit		Pied flycatcher	
	F_df_	p	F_df_	p	F_df_	p
Fledging success*						
PC1_M_	**7.16**_**1, 29**_ **est.** − 0.55, **SE** 0.20	**0.012**	**5.11**_**1, 19**_ **est.** − 0.84, **SE** 0.37	**0.036**	**0.66**_**1, 28**_	**0.425**
Body mass	3.48_1, 28_	0.072	1.99_1, 18_	0.175	1.56_1, 27_	0.223
Temperature	0.09_1, 24_	0.770	**4.47**_**1, 19**_ **est.** 0.26, **SE** 0.12	**0.048**	0.50_1, 26_	0.488
PC1_B_	0.09_1, 25_	0.772	0.02_1, 17_	0.888	0.04_1, 24_	0.849
PC2_B_	0.82_1, 26_	0.373	**5.95**_**1, 19**_ **est.** 0.71, **SE** 0.29	**0.025**	0.03_1, 25_	0.859
Relative body mass**						
PC1_M_	**16.97**_**1, 29**_ **est.** − 12.11, **SE** 2.94	**0.0003**	**13.19**_**1, 24**_ **est.** − 12.71**, SE** 3.50	**0.001**	**3.12**_**1, 26**_	**0.089**
Temperature	0.10_1, 26_	0.759	1.26_1, 23_	0.274	0.02_1, 25_	0.901
PC1_B_	3.25_1, 27_ est. 1.97, SE 1.10	0.083	0.23_1, 18_	0.635	**20.01**_**1, 26**_ **est.** 2.87, **SE** 0.64	**0.0001**
PC2_B_	0.07_1, 25_	0.789	1.68_1, 19_	0.211	**4.80**_**1, 26**_ **est.** − 2.19, **SE** 1.0	**0.038**

^*^GLM (binomial distribution, logit link function)

^**^LM (Gaussian distribution, identity link function)

We further tested whether the bacterial families and genera that differed in their abundance between the study areas (DA analyses) showed any effects on fledging success or RBM. The families Rhodobacteraceae and Clostridiales Incertae Sedis XIII and genera *Anaerovorax*, *Catabacter* and *Eubacterium* had no significant association on fledging success or RBM in pied flycatchers (*p* > 0.05). In great tits, the genera *Catellicoccus* and *Serratia* had a negative association with fledging success (*Catellicoccus*: F_df_ = 11.65_1, 28_, *p* = 0.002, *Serratia*: F_df_ = 5.34_1, 28_, *p* = 0.028) and RBM (*Catellicoccus*: F_df_ = 8.65_1, 28_, *p* = 0.007, *Serratia*: F_df_ = 6.52_1, 28_, *p* = 0.016), while *Rhodococcus* had no significant effects on fledging success (*p* > 0.05), but showed positive association on RBM (F_df_ = 7.61_1, 28_, *p* = 0.010). *Arthrobacter* had no significant effects on fledging success or RBM of great tit nestlings (*p* > 0.05).

## Discussion

### Core gut microbiota in three passerine species

Firmicutes, Proteobacteria and Actinobacteria were the major gut bacterial phyla across our study species, consistent with earlier avian studies (Bodawatta et al. 2022a; Drobniak et al. 2022; Fu et al. 2020; Grond et al. 2018; Kim et al. 2023; Kropáčková et al. 2017; Liukkonen et al. 2023). Firmicutes, the most abundant phyla in great tits and pied flycatchers, play a crucial role in fermenting organic molecules to supply energy for the host (Flint et al. 2008; Turnbaugh et al. 2006), influence weight gain and fat storage in chickens (Angelakis and Raoult 2010) and mammals (Turnbaugh et al. 2006), and aid in metabolism, digestion, and protein absorption within the gut (Grond et al. 2018). In contrast, Proteobacteria dominated the blue tit gut microbiota. Despite the higher proportion of Proteobacteria in birds compared to mammals, their functions remain poorly understood. Nevertheless, Proteobacteria include several opportunistic pathogens like *Campylobacter*, *Escherichia*, *Rickettsia* and *Salmonella* (Diakou et al. 2016; Keller et al. 2011; Ryu et al. 2014; Wallmenius et al. 2014). They have been associated with metabolic and immune disorders as well as ecological imbalances within the gut thereby posing potential threats to host health (Colston and Jackson 2016). Actinobacteria, the third most abundant phylum across species, inhabit diverse environments, including terrestrial and marine habitats as well as gastrointestinal tracts. They include pathogens like *Mycobacterium*, but also commensal bacteria utilized as probiotics in animals (Barka et al. 2016; Grond et al. 2018; Kailasapathy and Chin 2000).

Bacterial orders Lactobacillales, Clostridiales, and Actinomycetales were found across all three species, with more species-specific variation observed at lower taxonomic levels. The genus *Clostridium *sensu stricto, known for containing significant human and animal pathogens (Li et al. 2023), was prevalent in all studied species, along with *Enterococcus* and *Diplorickettsia* in great tits and pied flycatchers. *Enterococci*, typically part of the natural gut flora and beneficial due to probiotic effects (Fisher and Phillips 2009, Kwit et al. 2023; Stępień-Pyśniak et al. 2018), can also exhibit opportunistic pathogenic behavior and resistance to antibiotics. Wild birds serve as important reservoirs of enterococci (Kwit et al. 2023), potentially facilitating microbial transfer between wild and domesticated animals (Ben Yahia et al. 2018; Marrow et al. 2009; Radhouani et al. 2012). *Lactobacillus*, also common in pied flycatchers, offers protection against various bacterial pathogens (Lee et al. 2020) by producing antimicrobial substances and aiding in detoxification processes (Hammes and Hertel 2006). It has shown efficacy in mitigating the toxic effects of metals such as lead (Pb) and Cd (George et al. 2021; Shi et al. 2017). *Ureaplasma*, prevalent in gastrointestinal and urogenital tracts of vertebrates (Kropáčková et al. 2017) and occasionally contributed to severe pathogenesis (Sumithra et al. 2013), was one of the most abundant bacterial genera in both tit species. *Catellicoccus*, which is frequently found in the guts of seabirds (Góngora et al. 2021) and some passerines (Benskin et al. 2010; Kreisinger et al. 2017) was the most abundant genus in great tit, whereas *Pseudomonas*, associated with several diseases in animals and plants, was common in blue tits (Abd El-Ghany 2021; Eraky et al. 2020). *Pseudomonas* tended to be more abundant in polluted areas, which may be due to its high resistance to heavy metals, such as Cd, Pb and As (Al-Ansari et al. 2021; Pramanik et al. 2021; Zhou et al. 2023), making them an interesting taxonomic group in polluted environments.

### Association between metals and gut bacteria

We found 11 taxa that were differentially abundant between the polluted and control areas. Candidatus Saccharibacteria was more abundant in polluted area compared to control area in blue tits. This highly ubiquitous phylum can be found in soils, sediments, wastewater and animals, including earthworms (Rattray et al. 2010), mice (Salzman et al. 2002), birds (Ma et al. 2023) and canines (Dewhirst et al. 2012). In pied flycatchers, Rhodobacteraceae, which has been shown to be more common in the digestive tract of insectivorous than omnivorous birds (Bodawatta et al. 2018), was more abundant in polluted areas, but did not show effects on fledging success or nestling growth (RBM). In contrast, Clostridiales Incertae Sedis XIII, *Anaerovorax*, *Catabacter,* and *Eubacterium* were more abundant in control areas compared to polluted areas. Similarly, these bacteria exhibited no adverse fitness effects on pied flycatchers. In great tits, *Serratia* and *Catellicoccus* were more abundant in polluted areas, and both were further associated with reduced fledging success and nestling growth. The finding suggest that these bacterial groups may tolerate metals and/or act as potential opportunistic pathogens. For *Serratia*, this effect may be linked to its potential pathogenicity in birds and mammals (Saidenberg et al. 2007), despite its relatively low abundance found in the gut. *Catellicoccus*, on the other hand, is also suggested to be advantageous to birds by improving their immune response and facilitating nutrient transport (Benskin et al. 2010), thereby aiding in the optimization of nutrition under severe conditions (Góngora et al. 2021). Opposite to them, *Arthrobacter* and *Rhodococcus*, commonly found in soil, water and plants, were more abundant in control areas. *Rhodococcus* was positively associated to growth in great tit nestlings, but neither *Rhodococcus* nor *Arthrobacter* associated with fledging success. Of these bacteria, most *Rhodococcus* strains have been found to have very high levels of metal resistance and are potential agent for the bioremediation of pollutants, including heavy metals (Nazari et al. 2022). However, the causal link between metal and microbiota interactions with growth is still unclear, but our results could suggest that pollution, through its effects on gut microbiota, may play a role in determining growth conditions. Furthermore, the function of these bacterial taxa in bird gut is poorly known, and future studies should consider combining metabolomics analyses with microbial data.

### Association of metals with gut bacterial diversity and composition

The alpha diversity measured as Shannon diversity index, Chao1 richness, and observed richness did not differ significantly between the polluted and control areas in any of our study species, suggesting no direct link to environmental pollution. However, the overall gut bacterial community composition (beta diversity) differed between the study areas. Reduced richness and changes in the composition of plumage bacteria have been previously reported in birds exposed to Pb (Chatelain et al. 2016), whereas birds exposed to Zn have exhibited lower bacterial loads and changes in both plumage- (Chatelain et al. 2016) and gastrointestinal bacteria community composition (Hojberg et al. 2005; Vahjen et al. 2010). Also, decreased alpha diversity of gut microbiota in tree sparrows (*Passer montanus*) inhabiting metal-polluted areas has been observed by Zhang et al. (2023). However, the effects of metals on microbiota are likely dose-dependent, which could explain the variation across different studies. For example, the Pb and Zn levels in our study area are relatively low compared to heavily polluted areas. Metal-related variation in bacterial communities was further examined at order level by using the principal components of the bacterial taxa. Only bacterial component PC1_B_ (including Acidimicrobiales, Actinomycetales, Gaiellales, Solirubrobacterales, Bacillales, Planctomycetales, Rhodobacterales) showed higher bacterial abundance with increased metal levels. It is possible that these bacterial taxa may tolerate or be resistant to metals and/or may have an increased capacity to detoxify metals (Li et al. 2016; Mawang et al. 2021; Nazari et al. 2022; Presentato et al. 2020), thus preventing their harmful effects. However, the causality between the metals and these microbes remains unknown and should be confirmed with experimental studies.

### Species-specific differences in bacterial communities

The pied flycatchers had a considerably different overall bacterial community composition compared to great tits and blue tits, while tit species shared a more similar composition. Pied flycatchers further showed clearly higher bacterial abundances of two bacterial components PC2_B_ (including Coriobacteriales, Bacteroidales, Clostridiales, Erysipelotrichales, Desulfovibrionales, Sphingomonadales) and PC4_B_ (including Chlamydiales, Legionellales and Entomoplasmatales) compared to both tit species. This may be linked to the different phenology and/or ecology (e.g., different diet and feeding habits or the different migratory behaviour) of the species. High individual variation has been observed in several migratory passerines (Lewis et al. 2016, 2017; Skeen et al. 2021) and also between migrants and their resident counterparts (Turjeman et al. 2020), potentially due to their exposure to new bacteria at their different breeding areas or stopover sites during migration (Turjeman et al. 2020). The species-specific differences in bacterial composition, like in PC4_B_ consisting mainly pathogenic bacteria, may also support the idea that migratory birds, such as pied flycatchers, harbour a higher abundance of pathogens with potentially higher infection intensity compared to resident species (Clark et al. 2016; Koprivnikar and Leung 2015; Leung and Koprivnikar 2016).

### Association of gut bacteria with life-history traits and temperature

The Shannon diversity index was negatively associated with brood size in great tits and blue tits, which could be related to higher within-brood competition, for example in the case of limited food resources and space or food quality leading to higher stress, as suggested by Somers et al. (2023). The availability of good-quality insect food is inferior in the polluted area (Eeva et al. 2005), which may reflect the lower alpha diversity of larger broods due to higher food competition. Some other studies have not found a similar association between brood size and microbial alpha diversity in great tits (Liukkonen et al. 2023; Somers et al. 2023), although brood size has been shown to correlate negatively with Proteobacteria abundance, and positively with Firmicutes abundance (Somers et al. 2023).

We further showed that the growth of the pied flycatchers was positively associated with Shannon diversity and bacterial richness. This suggests that heavier nestlings exhibited increased microbial diversity and richness possibly due to their better capacity to gain and/or maintain higher microbial diversity during their developmental stages. *Enterococcus*, for example, have been shown to correlate positively with nestling growth in pied flycatcher (Moreno et al. 2003) and this genus indeed was one of the most common in pied flycatchers in the present study. However, such diversity association was not seen in great tits or blue tits, which may be partly explained by their different diet (mainly caterpillars during the nestling phase) compared to pied flycatchers, which also use flying insects as food source for their nestlings. Heavier nestlings are typically found in control areas with better food availability compared to polluted areas (Eeva et al. 2005). However, as we did not see significant diversity differences between the areas, the higher alpha diversity of heavier nestlings may be influenced by some other environmental factors, such as diet quality or weather, which may affect the condition of the host via changes in gut bacteria composition. On the other hand, both fitness measures, fledging success and nestling growth (RBM), decreased with higher metal levels (PC1_M_) in great tits and blue tits, but not in the pied flycatchers, indicating that pied flycatchers may be less sensitive to metal pollution either directly or indirectly via environmental changes compared to tit species. Diverse gut communities may be resistant to pathogens and less susceptible to environmental perturbation (Buffie and Pamer 2013), while lower microbial diversity is often considered detrimental to host species (Le Chatelier et al. (Le Chatelier, et al., 2013)), potentially leading to reduced nutrient assimilation or immunodeficiency (Teyssier et al. 2018). Similar to alpha diversity, also the abundances of bacteria belonging to PC1_B_ were clearly higher in well growing broods, whereas bacterial abundances of PC2_B_ increased in relation to reduced growth. The results suggest that PC1_B_ includes some beneficial bacteria for nestling growth, whereas potentially pathogenic bacteria, like Clostridiales in PC2_B_, may retard nestling growth. Our results are in accordance with the study of Teyssier et al. (2018), who showed that the microbial diversity increased with nestling body mass. Body condition of chicks is important for their survival especially after fledging (Monrós et al. 2002; Rodríguez et al. 2016; Tinbergen and Boerlijst 1990) and the role of microbiota may be highlighted at this point of nestling development (Teyssier et al. 2018).

The fledging success was enhanced by warmer temperature in blue tits, but not in the other species. Likewise, the Shannon diversity was linked to increased temperature inside the nest box only in blue tits, suggesting some temperature-derived differences in bacterial taxa. Some bacteria may favour warmer growth conditions thus benefiting higher ambient temperatures. Temperature inside the nest box further contributed to the variation in gut bacterial composition, for example warmer temperature was associated with increased abundance of bacteria belonging to PC1_B_, but decreased abundance of bacteria belonging to PC2_B_. In general, endotherms maintain a relatively constant body temperature, less influenced by environmental condition (Crompton et al. 1978). However, the thermoregulation of newly hatched chicks is not fully developed (Dawson and Evans 1960; Howell 1964; Price and Dzialowski 2018) limiting their ability to conserve heat (Dawson and Evans 1960; Rodríguez and Barba 2016), which could increase the impact of temperature on their gut microbiota. Environmental temperature is known to modify the gut microbiome of animals (Sepulveda and Moeller 2020; Tian et al. 2020). Especially Firmicutes have been shown to be sensitive to temperature variation; their relative abundances and alpha diversity have decreased with increased temperature in both endothermic and ectothermic host species (Bestion et al. 2017, Fontaine et al. (Fontaine, et al., 2018); Kohl and Yahn 2016, Zhu et al. 2019). The mechanism behind the association of temperature and bacteria is unclear, but may be related to the host metabolism and energetics to cope with thermal stress (Sepulveda and Moeller 2020; Sokolova et al. (Sokolova 2012)), potentially affecting the host investment in regulating microbiome composition.

## Conclusions

The gut bacterial taxa exhibited similarities across the bird species, although greater variation was observed at the genus level compared to higher taxa. The gut bacterial community composition of pied flycatchers differed from great tits and blue tits, while tit species were more alike. This suggests that factors such as diet, phenology, breeding habits, and the migratory status of the birds likely influence the distinct gut microbiota of pied flycatchers. Although metal concentrations were not directly associated with bacterial alpha diversity, certain bacterial taxa appeared to better tolerate increased metal concentrations, being more abundant in polluted areas compared to control areas. These observations may be linked to the enhanced metal tolerance or detoxification capacities of these bacterial orders within the host organisms. Notably, *Catellicoccus* and *Serratia* that appeared in higher numbers in polluted environments, were further associated with reduced fledging success in great tit nestlings. Additionally, gut bacteria were linked to the brood size, nestling growth, and temperature inside the nest boxes, indicating that gut microbiota is not only affected by environmental pollution but also several other factors, highlighting the complex host-microbe interactions. To understand the complex interactions between the host microbiota and various environmental factors, experimental studies would be necessary. However, our results provide evidence of metal-induced effects on the bacterial community composition of nestlings in a species-specific manner either directly or indirectly via diet or other environmental changes.

## Supplementary Information

Below is the link to the electronic supplementary material.

ESM 1 (DOCX 2.38 MB)

## Data Availability

Data used in this manuscript can be provided by the authors upon request.
