# Supplementary material for: Metal-induced changes in the early-life gut microbial composition and life-history traits in three insectivorous passerines
Source: Environ Sci Pollut Res Int. 2025 Nov 21;32(47):27068–85. doi: 10.1007/s11356-025-37181-x (PMC12675715; doi:10.1007/s11356-025-37181-x)
Supplement: Supplementary file 1 — (DOCX 2.38 MB) [file 11356_2025_37181_MOESM1_ESM.docx]

**Supplementary material for**

Rainio et al. 2025. Metal-induced changes in the early-life gut microbial composition and life-history traits in three insectivorous passerines

Table of Contents

[1. Molecular analysis 1](#_Toc164780652)

[2. Bioinformatics 3](#_Toc164780653)

[3. Figures 4](#_Toc164780654)

Figure S1 …………………………………………………………………………………………………... 4

[Figure S2. 5](#_Toc164780655)

[4. Tables 5](#_Toc164780656)

[Table S1. 5](#_Toc164780657)

[Table S2. 7](#_Toc164780658)

[Table S3. 8](#_Toc164780659)

[A. Blue tit (Cyanistes caeruleus) 8](#_Toc164780660)

[B. Great tit (*Parus major*) 10](#_Toc164780661)

[C. Pied flycatcher (*Ficedula hypoleuca*) 12](#_Toc164780662)

# Molecular analysis

All molecular work including DNA extraction, NGS library preparation, and sequencing as well as bioinformatics were carried out as turnkey service by DNA analysis company Bioname (Turku, Finland; www.bioname.fi). In summary, bacterial DNA was extracted from the faecal samples using Quick-DNA Fecal/Soil microbe Miniprep Kit (Zymo Research). Microbial 16S rRNA gene region (V4 region) was amplified using one primer pair: forward primer 515FB (GTGYCAGCMGCCGCGGTAA, see Parada et al., 2016; Walters et al., 2016) and reverse primer 806RB (GGACTACNVGGGTWTCTAAT, see Apprill et al., 2015; Caporaso et al., 2011). These primers are designed to amplify a highly variable, short gene region to enable bacterial taxa identification. All the primers included a linker-tag to enable the subsequent attachment of NGS adapters. To increase the amplicon library diversity, each primer was used as four different versions, so that they included 0-4 heterogeneity spacer nucleotides between the linker-tag and the actual locus-specific oligo. All PCR reactions were carried out as two technical replicates and each replicate contained two heterogeneity versions of each primer. Negative and blank controls were added to each PCR batch to measure the purity of reagents and the level of cross-contamination. For the PCR reaction we used 5µl of 2 × MyTaq™ HS Red Mix (Bioline, UK), 2.4µl of sterile MQ-H_2_O, 150nM of each primer (two forward and two reverse primer versions), and 2µl of DNA extract per each sample in a 10µl reaction volume. The PCR amplification was performed as follows: 3 min denaturation at 95°C followed by 35 cycles of denaturing at 95°C for 45 sec, annealing at 50°C for 1 min and extension at 72°C for 1 min 30 sec. The final extension was carried out at 72°C for 10 min. Libraries for sequencing were prepared by using dual indexing strategy in a library PCR reaction, where each reaction was prepared with a unique combination of forward and reverse index primers (i5 and i7). For a reaction volume of 10µl, we used 5µl of 2 × MyTaq HS Red Mix, 500nM of each tagged and indexed primer (i5 and i7) and 3µl of locus-specific PCR product (from first PCR). The second PCR was performed as follows: initial denaturation for 3 min at 98°C followed by 12 cycles of denaturing at 95°C for 20 sec, annealing at 60°C for 15 sec and extension at 72°C for 30 sec, followed by 3 min at 72°C. All indexed reactions were then pooled in equal volumes, separately for each primer pair and replicate, and purified using dual-SPRI (solid-phase reversible immobilization) purification protocol (see more detailed description in Vesterinen et al., 2018; Vesterinen et al., 2016). The sequencing was performed on the Illumina NovaSeq6000 SP Flowcell using 2 x 250 bp paired-end read length (Illumina Inc. San Diego, California, USA) in the Finnish Functional Genomics Centre (FFGC, University of Turku, Finland). The sequencing run included a small amount of PhiX control library.

# Bioinformatics

The demultiplexed reads assigned to each original sample were uploaded to CSC servers (IT Center for Science, Finland) for trimming and further analysis. Paired-end reads were merged and trimmed for quality using 64-bit VSEARCH version 2.14.2. (Rognes et al., 2016) with the command ´fastq_mergepairs´. Primers were removed from the merged reads with CUTADAPT version 2.7 (Martin, 2011) software with 20 % rate for primer mismatches and 100 bp minimum length. The reads were then collapsed into unique sequences (singletons removed) using VSEARCH command ‘fastx_uniques’, denoised (=chimeras removed) and reads were clustered into ZOTUs (zero-radius OTUs) with command ‘*unoise3’* (default options: minsize=8 and alpha=2) using 32-bit USEARCH version 11 (Edgar, 2010). ZOTUs do not practically differ from traditional clustering of OTUs (which are based on pre-set percentage threshold), but the UNOISE algorithm performs better in removing a) chimeras, b) PhiX sequences and c) Illumina artefacts according to Edgar and Flyvbjerg (2015). ZOTUs were mapped back to the primer-trimmed reads to construct a zotutable using VSEARCH ‘usearch_global’ algorithm. The initial ZOTU-table consisted 32,001,435 reads. The sequence variants were assigned to taxa using custom database (rdp_16s_v16.fa; available from https://www.drive5.com/usearch/manual/sintax_downloads.html) with SINTAX (Edgar, 2010) in VSEARCH (Rognes et al., 2016). These reads were subject to further filtering, after which we identified 17,839 out of 19,469 ZOTUs (91.6%) and 29,320,908 reads (95.8%) before final filtering. The data was then collapsed per taxonomic assignations, so that all reads assigned similar per each taxon were summarised within a sample. To analyse the two PCR replicates generally, the reads assigned to the same taxa in each replicate per sample were plotted against each other or by taxa. To remove the reads that could have been misassigned during index demultiplexing (known as ‘tag jumping’ or ‘sample crosstalk’), we used a fixed tag-jumping rate of 0.05%. Finally, reads were filtered for non-target reads, such as Chloroplast DNA. Low abundance ZOTU’s (= read count less than 2) were removed from the data. After filtering, majority of the reads were retained in the data, including 19,208,556 reads (~ 220,788 reads per sample).

# Figures

Figure S1. Rarefaction curves for the unfiltered data (R-package vegan) for A) all samples included, and PCR replicates merged using average number of reads across replicates (truncated to integers) and B) samples with less than 100,000 reads included, and PCR replicates merged using average number of reads across replicates (truncated to integers), C) only replicate 1 included, D) only samples with less than 100,000 reads from replicate 1 included, E) only replicate 2 included, F) only samples with less than 100,000 reads from replicate 2 included. Blue lines represent the number of rarefied ZOTUs along the number of reads in each sample. When all samples included, the curves flatten along the number of reads even in small library sizes, indicating that no considerable amount of new ZOTUs would have been found if the number of reads were higher. The red dashed line represents the rarefaction depth used in the main analyses.


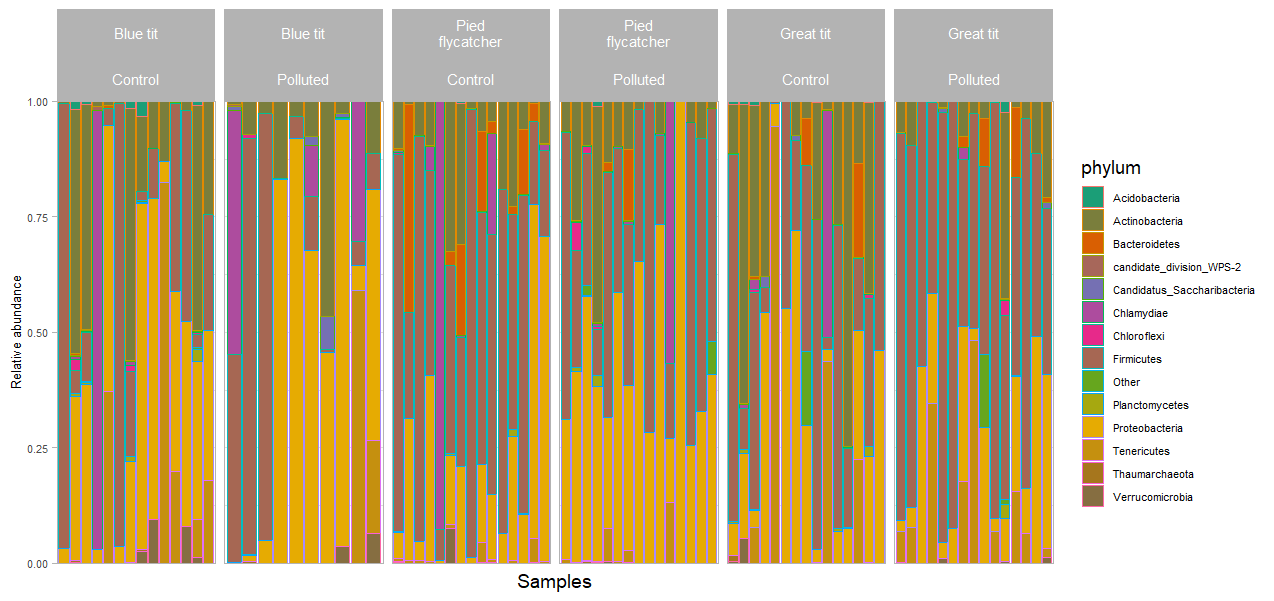


Figure S2. Relative abundance of the bacteria at phylum level in blue tit (*Cyanistes caeruleus*), great tit (*Parus major*) and pied flycatcher (*Ficedula hypoleuca*) nestlings in polluted and control areas. Taxa with the prevalence threshold of <10% across all samples were assigned to “Other”. Analyses were performed with samples rarefied to read depth of 29 000.

# Tables

| Table S1. The most abundant (%) faecal gut bacteria in great tits (*Parus major*), blue tits (*Cyanistes caeruleus*) and pied flycatcher (*Ficedula hypoleuca*) at phylum, order and genus level. | | | | | |
| --- | --- | --- | --- | --- | --- |
| **Blue tit** | | **Great tit** | | **Pied flycatcher** | |
| **Phylum** | **mean** | **Phylum** | **mean** | **Phylum** | **mean** |
| Proteobacteria | 37.90 | Firmicutes | 46.72 | Firmicutes | 43.68 |
| Firmicutes | 25.62 | Proteobacteria | 21.51 | Proteobacteria | 33.35 |
| Actinobacteria | 15.55 | Actinobacteria | 15.14 | Actinobacteria | 10.21 |
| Tenericutes | 10.19 | Tenericutes | 10.49 | Chlamydiae | 6.05 |
| Chlamydiae | 7.97 | Bacteroidetes | 2.03 | Bacteroidetes | 4.18 |
| Verrucomicrobia | 1.34 | Chlamydiae | 1.84 | Tenericutes | 1.29 |
| Candidatus_Saccharibacteria | 0.46 | Synergistetes | 1.06 | Verrucomicrobia | 0.30 |
| Acidobacteria | 0.34 | Verrucomicrobia | 0.30 | Chloroflexi | 0.29 |
| Planctomycetes | 0.23 | Planctomycetes | 0.26 | Fusobacteria | 0.27 |
| Chloroflexi | 0.21 | Candidatus_Saccharibacteria | 0.22 | Planctomycetes | 0.17 |
| candidate_division_WPS-2 | 0.06 | Acidobacteria | 0.18 | Acidobacteria | 0.07 |
| Bacteroidetes | 0.05 | Chloroflexi | 0.17 | Synergistetes | 0.05 |
| Thaumarchaeota | 0.05 | Thaumarchaeota | 0.05 | Thaumarchaeota | 0.04 |
| Gemmatimonadetes | 0.03 | Armatimonadetes | 0.03 | Candidatus_Saccharibacteria | 0.03 |
| Armatimonadetes | <0.01 | Euryarchaeota | 0.01 | Gemmatimonadetes | <0.01 |
| Deinococcus-Thermus | <0.01 | candidate_division_WPS-2 | 0.01 | candidate_division_WPS-2 | <0.01 |
| Fibrobacteres | <0.01 | Deinococcus-Thermus | 0.01 | Armatimonadetes | <0.01 |
|  |  | Gemmatimonadetes | <0.01 | Deferribacteres | <0.01 |
|  |  | candidate_division_WPS-1 | <0.01 | candidate_division_WPS-1 | <0.01 |
|  | | | | | |
| **order** | **Mean** | **order** | **Mean** | **order** | **Mean** |
| Lactobacillales | 15.79 | Lactobacillales | 28.76 | Clostridiales | 22.52 |
| Enterobacteriales | 12.12 | Clostridiales | 13.67 | Lactobacillales | 18.30 |
| Actinomycetales | 11.96 | Actinomycetales | 13.15 | Legionellales | 16.14 |
| Mycoplasmatales | 9.44 | Mycoplasmatales | 8.82 | Actinomycetales | 8.65 |
| Clostridiales | 8.04 | Enterobacteriales | 4.79 | Enterobacteriales | 8.41 |
| Chlamydiales | 7.50 | Legionellales | 4.54 | Chlamydiales | 6.05 |
| Pseudomonadales | 5.59 | Rhizobiales | 3.13 | Rhizobiales | 2.79 |
| Rhizobiales | 5.32 | Pseudomonadales | 2.56 | Bacteroidales | 2.76 |
| Rickettsiales | 3.87 | Bacillales | 2.38 | Erysipelotrichales | 1.47 |
| Legionellales | 2.88 | NA | 2.33 | Rhodobacterales | 1.42 |
| Caulobacterales | 2.29 | Chlamydiales | 1.78 | Cytophagales | 1.30 |
| Solirubrobacterales | 2.25 | Entomoplasmatales | 1.43 | Pseudomonadales | 1.21 |
| Sphingomonadales | 1.66 | Erysipelotrichales | 1.29 | NA | 0.95 |
| Burkholderiales | 1.49 | Solirubrobacterales | 1.23 | Mycoplasmatales | 0.90 |
| NA | 1.32 | Desulfovibrionales | 1.22 | Solirubrobacterales | 0.85 |
| Bacillales | 1.23 | Flavobacteriales | 1.17 | NA | 0.65 |
| Xanthomonadales | 0.84 | Synergistales | 1.06 | Bacillales | 0.61 |
| Gaiellales | 0.78 | Burkholderiales | 0.76 | Coriobacteriales | 0.56 |
| Rhodospirillales | 0.75 | Bacteroidales | 0.72 | Rhodospirillales | 0.54 |
| Entomoplasmatales | 0.75 | Sphingomonadales | 0.70 | Desulfovibrionales | 0.48 |
|  | | | | | |
| **Genus** | **Mean** | **Genus** | **Mean** | **Genus** | **Mean** |
| Ureaplasma | 8.18 | Catellicoccus | 13.04 | Diplorickettsia | 16.08 |
| NA | 6.75 | Ureaplasma | 8.08 | Clostridium_sensu_stricto | 15.75 |
| Clostridium_sensu_stricto | 6.48 | Clostridium_sensu_stricto | 6.61 | Lactobacillus | 5.85 |
| Buchnera | 6.16 | Enterococcus | 4.75 | Enterococcus | 5.34 |
| Catellicoccus | 5.27 | NA | 4.04 | NA | 4.41 |
| Pseudomonas | 3.96 | Diplorickettsia | 3.56 | Escherichia/Shigella | 3.37 |
| NA | 3.88 | Escherichia/Shigella | 3.36 | NA | 3.33 |
| NA | 3.54 | NA | 3.28 | NA | 3.08 |
| Rickettsia | 3.46 | Arthrobacter | 3.04 | NA | 2.69 |
| Leifsonia | 2.92 | Lactobacillus | 2.56 | NA | 2.08 |
| Enterococcus | 2.60 | Pseudomonas | 2.21 | NA | 1.55 |
| Salinibacterium | 2.25 | Salinibacterium | 1.96 | Paracoccus | 1.38 |
| Lactobacillus | 2.08 | Carnobacterium | 1.93 | NA | 1.31 |
| Brevundimonas | 2.01 | NA | 1.70 | Serratia | 1.27 |
| Neochlamydia | 1.98 | Clostridium_XlVa | 1.68 | Rothia | 1.25 |
| Mycobacterium | 1.88 | NA | 1.45 | Clostridium_XVIII | 1.16 |
| Diplorickettsia | 1.64 | Spiroplasma | 1.35 | Vagococcus | 1.10 |
| Sphingomonas | 1.19 | Sphingomonas | 1.30 | Dysgonomonas | 1.09 |
| NA | 1.18 | NA | 1.28 | NA | 0.94 |
| Spartobacteria_genera_incertae_sedis | 1.17 | Desulfovibrio | 1.19 | Methylobacterium | 0.93 |

| Table S2. Five differential abundance (DA) estimators ALDEx2, ANCOM-BC2, Corncob, DESeq2 and LinDA were used to identify the bacteria associated with study area (polluted and control) at different taxonomic levels (phylum, order, family, genus) in great tits (*Parus major*), blue tits (*Cyanistes caeruleus*) and pied flycatchers (*Ficedula hypoleuca*). The responses of bacterial taxa in which three or more of the five estimators indicated a significant response were considered significant. Non-rarefied observed abundances were used as input and the estimators queried at a significance level of p < 0.05 after Benjamini-Hochberg adjustment. The log2-fold changes (with upper and lower limits for 95% CI) correspond to effect sizes. Prior to the DA analyses the prevalence threshold was set to 10% for all samples. | | | | | | | | | |
| --- | --- | --- | --- | --- | --- | --- | --- | --- | --- |
| **Species** | **DA estimator** | **Level** | **Taxon** | **Log2FC** | **Lower 95%** | **Upper 95%** | **SE** | **Adj p** | **Elevated in** |
| *C. caeruleus* | LinDA | Phylum | Candidatus_Saccharibacteria | 5.02 | 1.88 | 8.15 | 1.52 | 0.041 | polluted |
| *C. caeruleus* | DESeq2 | Phylum | Candidatus_Saccharibacteria | 7.15 | 3.96 | 10.34 | 1.63 | 0.000 | polluted |
| *C. caeruleus* | Corncob | Phylum | Candidatus_Saccharibacteria | 5.45 | 2.10 | 8.79 | 1.12 | 0.005 | polluted |
| *F. hypoleuca* | LinDA | Order | Rhodobacterales | 4.86 | 1.85 | 7.87 | 1.47 | 0.045 | polluted |
| *F. hypoleuca* | DESeq2 | Order | Rhodobacterales | 8.83 | 5.82 | 11.84 | 1.53 | 0.000 | polluted |
| *F. hypoleuca* | ANCOM-BC2 | Order | Rhodobacterales | 3.29 | 1.12 | 5.45 | 1.10 | 0.046 | polluted |
| *F. hypoleuca* | Corncob | Order | Rhodobacterales | 10.92 | 7.75 | 14.10 | 1.07 | 0.000 | polluted |
| *F. hypoleuca* | LinDA | Family | *Clostridiales_Incertae_Sedis_XIII* | -4.20 | -6.13 | -2.26 | 0.95 | 0.005 | control |
| *F. hypoleuca* | DESeq2 | Family | *Clostridiales_Incertae_Sedis_XIII* | -7.08 | -11.09 | -3.08 | 2.04 | 0.006 | control |
| *F. hypoleuca* | ANCOM-BC2 | Family | *Clostridiales_Incertae_Sedis_XIII* | -2.53 | -3.84 | -1.23 | 0.67 | 0.004 | control |
| *F. hypoleuca* | LinDA | Family | *Rhodobacteraceae* | 5.01 | 1.95 | 8.07 | 1.50 | 0.036 | polluted |
| *F. hypoleuca* | DESeq2 | Family | *Rhodobacteraceae* | 8.14 | 5.03 | 11.25 | 1.59 | 0.000 | polluted |
| *F. hypoleuca* | ANCOM-BC2 | Family | *Rhodobacteraceae* | 3.27 | 1.22 | 5.33 | 1.05 | 0.033 | polluted |
| *F. hypoleuca* | Corncob | Family | *Rhodobacteraceae* | 10.80 | 7.71 | 13.89 | 1.04 | 0.000 | polluted |
| *F. hypoleuca* | LinDA | Genus | *Anaerovorax* | -3.97 | -6.00 | -1.94 | 0.99 | 0.026 | control |
| *F. hypoleuca* | DESeq2 | Genus | *Anaerovorax* | -24.89 | -29.31 | -20.47 | 2.25 | 0.000 | control |
| *F. hypoleuca* | ANCOM-BC2 | Genus | *Anaerovorax* | -2.42 | -3.75 | -1.08 | 0.68 | 0.021 | control |
| *F. hypoleuca* | LinDA | Genus | *Catabacter* | -3.15 | -4.71 | -1.58 | 0.76 | 0.026 | control |
| *F. hypoleuca* | DESeq2 | Genus | *Catabacter* | -5.77 | -10.00 | -1.54 | 2.16 | 0.031 | control |
| *F. hypoleuca* | ANCOM-BC2 | Genus | *Catabacter* | -1.85 | -2.82 | -0.87 | 0.50 | 0.018 | control |
| *F. hypoleuca* | LinDA | Genus | *Eubacterium* | -5.84 | -8.31 | -3.37 | 1.21 | 0.010 | control |
| *F. hypoleuca* | DESeq2 | Genus | *Eubacterium* | -8.92 | -12.18 | -5.65 | 1.67 | 0.000 | control |
| *F. hypoleuca* | ANCOM-BC2 | Genus | *Eubacterium* | -3.52 | -5.11 | -1.92 | 0.81 | 0.004 | control |
| *F. hypoleuca* | Corncob | Genus | *Eubacterium* | -10.51 | -13.61 | -7.41 | 1.05 | 0.000 | control |
| *P. major* | LinDA | Genus | *Arthrobacter* | -5.34 | -8.50 | -2.17 | 1.55 | 0.048 | control |
| *P. major* | DESeq2 | Genus | *Arthrobacter* | -3.80 | -6.77 | -0.82 | 1.52 | 0.043 | control |
| *P. major* | Corncob | genus | *Arthrobacter* | -6.61 | -9.68 | -3.54 | 1.04 | 0.001 | control |
| *P. major* | LinDA | genus | *Catellicoccus* | 8.08 | 3.37 | 12.80 | 2.31 | 0.048 | polluted |
| *P. major* | DESeq2 | genus | *Catellicoccus* | 15.01 | 9.96 | 20.07 | 2.58 | 0.000 | polluted |
| *P. major* | ANCOM-BC2 | genus | *Catellicoccus* | 5.45 | 2.42 | 8.49 | 1.55 | 0.023 | polluted |
| *P. major* | LinDA | genus | *Rhodococcus* | -4.60 | -7.20 | -2.00 | 1.27 | 0.046 | control |
| *P. major* | DESeq2 | genus | *Rhodococcus* | -6.35 | -9.64 | -3.07 | 1.68 | 0.001 | control |
| *P. major* | Corncob | genus | *Rhodococcus* | -6.89 | -10.62 | -3.15 | 1.26 | 0.003 | control |
| *P. major* | LinDA | genus | *Serratia* | 3.56 | 1.68 | 5.44 | 0.92 | 0.031 | polluted |
| *P. major* | DESeq2 | genus | *Serratia* | 23.55 | 18.52 | 28.58 | 2.57 | 0.000 | polluted |
| *P. major* | ANCOM-BC2 | genus | *Serratia* | 2.30 | 1.12 | 3.48 | 0.60 | 0.011 | polluted |

| Table S3. Five differential abundance (DA) estimators ALDEx2, ANCOM-BC2, Corncob, DESeq2 and LinDA were used to identify the bacteria associated with study area (polluted and control) at different taxonomic levels (phylum, order, family, genus) in A) blue tits (*Cyanistes caeruleus*), B) great tits (*Parus major*) and C) pied flycatchers (*Ficedula hypoleuca*). Non-rarefied observed abundances were used as input and the estimators queried at a significance level of p < 0.05 after Benjamini-Hochberg adjustment. The log2-fold changes (with upper and lower limits for 95% CI) correspond to effect sizes. Prior to the DA analyses the prevalence threshold was set to 10% for all samples. | | | | | | | | |
| --- | --- | --- | --- | --- | --- | --- | --- | --- |
| A. Blue tit (Cyanistes caeruleus) | | | | | | | | |
| **DA tool** | **Level** | **Taxon** | **Log2FC** | **Lower** | **Upper** | **SE** | **Adj p** | **Elevated in** |
| DESeq2 | family | Acetobacteraceae | -5.7360 | -9.8182 | -1.6539 | 2.0827 | 0.0399 | clean |
| Corncob | family | Acetobacteraceae | -8.9080 | -12.6465 | -5.1695 | 1.2527 | 0.0000 | clean |
| Corncob | family | Acidimicrobiaceae | -7.7012 | -13.6844 | -1.7180 | 2.0048 | 0.0447 | clean |
| Corncob | family | Acidimicrobineae_incertae_sedis | -5.9622 | -10.5311 | -1.3934 | 1.5309 | 0.0447 | clean |
| Corncob | phylum | Acidobacteria | -6.1558 | -9.6426 | -2.6690 | 1.1683 | 0.0034 | clean |
| Corncob | class | Acidobacteria_Gp16 | -6.1128 | -10.5245 | -1.7011 | 1.4782 | 0.0291 | clean |
| DESeq2 | genus | Actinoplanes | -8.3471 | -13.4052 | -3.2889 | 2.5807 | 0.0077 | clean |
| DESeq2 | genus | Aeromicrobium | -5.7003 | -10.0606 | -1.3401 | 2.2246 | 0.0416 | clean |
| Corncob | genus | Aeromicrobium | -8.6554 | -14.4231 | -2.8877 | 1.9326 | 0.0330 | clean |
| Corncob | family | Alcaligenaceae | -6.0693 | -10.7763 | -1.3623 | 1.5772 | 0.0447 | clean |
| DESeq2 | genus | Aquabacterium | 28.6173 | 22.8335 | 34.4011 | 2.9509 | 0.0000 | polluted |
| Corncob | genus | Aquamicrobium | -6.4877 | -10.8744 | -2.1009 | 1.4699 | 0.0330 | clean |
| DESeq2 | genus | Aquicella | 6.3426 | 2.7300 | 9.9552 | 1.8431 | 0.0044 | polluted |
| DESeq2 | genus | Aquihabitans | -22.2881 | -28.0984 | -16.4778 | 2.9645 | 0.0000 | clean |
| DESeq2 | genus | Aquisphaera | -23.2233 | -28.8801 | -17.5665 | 2.8861 | 0.0000 | clean |
| Corncob | family | Beijerinckiaceae | -6.6802 | -11.5899 | -1.7704 | 1.6451 | 0.0423 | clean |
| Corncob | class | Betaproteobacteria | 2.6469 | 0.67164 | 4.62213 | 0.6618 | 0.0292 | polluted |
| DESeq2 | genus | Bosea | -7.0663 | -11.7431 | -2.3895 | 2.3861 | 0.0143 | clean |
| Corncob | genus | Bosea | -8.6140 | -12.8285 | -4.3994 | 1.4122 | 0.0019 | clean |
| DESeq2 | genus | Buchnera | 10.9813 | 6.1301 | 15.8324 | 2.4751 | 0.0001 | polluted |
| Corncob | genus | Burkholderia | -8.8740 | -13.4486 | -4.2994 | 1.5328 | 0.0033 | clean |
| Corncob | family | Burkholderiaceae | -8.9115 | -13.6625 | -4.1606 | 1.5919 | 0.0026 | clean |
| Corncob | order | Burkholderiales | 2.7769 | 0.6878 | 4.8660 | 0.7000 | 0.0328 | polluted |
| DESeq2 | family | Burkholderiales_incertae_sedis | 28.0301 | 22.2464 | 33.8139 | 2.9509 | 0.0000 | polluted |
| LinDA | phylum | Candidatus_Saccharibacteria | 5.0152 | 1.8775 | 8.1529 | 1.5235 | 0.0415 | polluted |
| DESeq2 | phylum | Candidatus_Saccharibacteria | 7.1506 | 3.9588 | 10.3425 | 1.6285 | 0.0001 | polluted |
| Corncob | phylum | Candidatus_Saccharibacteria | 5.4473 | 2.1037 | 8.7908 | 1.1203 | 0.0049 | polluted |
| DESeq2 | genus | Carnobacterium | -7.3960 | -12.5379 | -2.2540 | 2.6235 | 0.0213 | clean |
| DESeq2 | phylum | Chlamydiae | 8.1522 | 4.6167 | 11.6877 | 1.8038 | 0.0001 | polluted |
| DESeq2 | class | Chlamydiia | 8.4993 | 4.30953 | 12.6891 | 2.1376 | 0.0016 | polluted |
| DESeq2 | order | Chlamydiales | 6.9704 | 2.7678 | 11.1729 | 2.1442 | 0.0142 | polluted |
| Corncob | family | Conexibacteraceae | -5.7245 | -10.2904 | -1.1587 | 1.5299 | 0.0465 | clean |
| Corncob | family | Corynebacteriaceae | 7.6466 | 3.0322 | 12.2611 | 1.5462 | 0.0085 | polluted |
| DESeq2 | genus | Corynebacterium | 7.7682 | 2.7746 | 12.7618 | 2.5477 | 0.0121 | polluted |
| Corncob | genus | Corynebacterium | 7.2345 | 2.7437 | 11.7253 | 1.5047 | 0.0185 | polluted |
| DESeq2 | family | Cryptosporangiaceae | -8.3452 | -12.9977 | -3.6927 | 2.3737 | 0.0045 | clean |
| DESeq2 | genus | Enterococcus | -5.1964 | -8.5809 | -1.8118 | 1.7268 | 0.0129 | clean |
| Corncob | family | Erysipelotrichaceae | -6.0095 | -9.9624 | -2.0567 | 1.3245 | 0.0181 | clean |
| Corncob | order | Erysipelotrichales | -5.9309 | -9.9747 | -1.8871 | 1.3550 | 0.0159 | clean |
| Corncob | class | Erysipelotrichia | -5.9012 | -9.96076 | -1.8416 | 1.3602 | 0.0277 | clean |
| Corncob | genus | Escherichia/Shigella | 10.1854 | 5.8794 | 14.4914 | 1.4428 | 0.0002 | polluted |
| DESeq2 | genus | Jatrophihabitans | -24.5643 | -29.9944 | -19.1341 | 2.7705 | 0.0000 | clean |
| DESeq2 | family | Kineosporiaceae | -22.4536 | -27.5992 | -17.3080 | 2.6253 | 0.0000 | clean |
| Corncob | family | Labilitrichaceae | -6.9842 | -12.5042 | -1.4642 | 1.8496 | 0.0457 | clean |
| DESeq2 | genus | Labrys | -23.1537 | -28.8106 | -17.4968 | 2.8862 | 0.0000 | clean |
| DESeq2 | family | Lactobacillaceae | 10.8405 | 6.1448 | 15.5363 | 2.3958 | 0.0001 | polluted |
| Corncob | family | Lactobacillaceae | 11.6955 | 8.3792 | 15.0117 | 1.1112 | 0.0000 | polluted |
| DESeq2 | genus | Lactobacillus | 11.6204 | 6.8961 | 16.3448 | 2.4104 | 0.0000 | polluted |
| Corncob | genus | Lactobacillus | 7.8522 | 3.6426 | 12.0619 | 1.4105 | 0.0046 | polluted |
| DESeq2 | genus | Legionella | 6.4024 | 2.4830 | 10.3219 | 1.9997 | 0.0077 | polluted |
| DESeq2 | family | Legionellaceae | 5.7560 | 2.1405 | 9.3715 | 1.8446 | 0.0157 | polluted |
| DESeq2 | order | Legionellales | 5.4481 | 2.8141 | 8.0822 | 1.3439 | 0.0019 | polluted |
| Corncob | family | Methylobacteriaceae | -7.1258 | -12.6371 | -1.6144 | 1.8467 | 0.0447 | clean |
| Corncob | genus | Methylobacterium | -8.4047 | -13.4183 | -3.3910 | 1.6799 | 0.0143 | clean |
| DESeq2 | family | Micromonosporaceae | -9.0639 | -13.7170 | -4.4108 | 2.3740 | 0.0016 | clean |
| Corncob | genus | Nocardia | -5.9606 | -10.0602 | -1.8610 | 1.3737 | 0.0330 | clean |
| DESeq2 | genus | Phycicola | -21.9893 | -27.7976 | -16.1810 | 2.9634 | 0.0000 | clean |
| DESeq2 | genus | Phyllobacterium | -22.1531 | -27.9639 | -16.3424 | 2.9647 | 0.0000 | clean |
| DESeq2 | family | Polyangiaceae | -21.9429 | -27.5205 | -16.3652 | 2.8458 | 0.0000 | clean |
| Corncob | order | Pseudomonadales | 3.4491 | 1.7836 | 5.1145 | 0.5580 | 0.0002 | polluted |
| Corncob | family | Pseudonocardiaceae | -6.3889 | -11.1166 | -1.6611 | 1.5841 | 0.0423 | clean |
| Corncob | genus | Reyranella | -9.7005 | -15.1847 | -4.2164 | 1.8376 | 0.0083 | clean |
| Corncob | family | Rhizobiaceae | -8.7738 | -14.9477 | -2.5999 | 2.0687 | 0.0322 | clean |
| DESeq2 | genus | Rhizobium | -7.0549 | -12.3508 | -1.7589 | 2.7020 | 0.0379 | clean |
| Corncob | family | Rhodospirillaceae | -9.0511 | -14.2940 | -3.8082 | 1.7567 | 0.0058 | clean |
| DESeq2 | order | Rhodospirillales | -7.0732 | -10.8433 | -3.3032 | 1.9235 | 0.0044 | clean |
| Corncob | order | Rhodospirillales | -9.2200 | -12.8286 | -5.6114 | 1.2091 | 0.0000 | clean |
| DESeq2 | genus | Rickettsia | -9.4851 | -15.2612 | -3.7090 | 2.9470 | 0.0077 | clean |
| DESeq2 | family | Rickettsiaceae | -8.3392 | -14.1014 | -2.5770 | 2.9399 | 0.0348 | clean |
| Corncob | family | Roseiarcaceae | -6.8786 | -11.3701 | -2.3871 | 1.5050 | 0.0181 | clean |
| DESeq2 | genus | Saccharibacteria_genera_incertae_sedis | 6.5973 | 3.0954 | 10.0993 | 1.7867 | 0.0019 | polluted |
| Corncob | genus | Saccharibacteria_genera_incertae_sedis | 5.2160 | 1.9636 | 8.4684 | 1.0898 | 0.0185 | polluted |
| Corncob | family | Solirubrobacteraceae | -6.6396 | -10.3171 | -2.9622 | 1.2322 | 0.0037 | clean |
| Corncob | order | Solirubrobacterales | -5.2844 | -8.4842 | -2.0847 | 1.0722 | 0.0052 | clean |
| Corncob | family | Sphingobacteriaceae | -5.8796 | -8.9812 | -2.7780 | 1.0393 | 0.0026 | clean |
| Corncob | order | Sphingobacteriales | -5.9847 | -8.8986 | -3.0708 | 0.9764 | 0.0002 | clean |
| Corncob | class | Sphingobacteriia | -5.9794 | -8.9009 | -3.0579 | 0.9789 | 0.0005 | clean |
| DESeq2 | order | Sphingomonadales | 3.7009 | 1.2120 | 6.1897 | 1.2698 | 0.0330 | polluted |
| Corncob | genus | Streptacidiphilus | -7.2653 | -12.2626 | -2.2681 | 1.6744 | 0.0330 | clean |
| DESeq2 | genus | Streptomyces | -7.0105 | -11.2129 | -2.8081 | 2.1441 | 0.0075 | clean |
| Corncob | family | Streptomycetaceae | -5.5787 | -9.9720 | -1.1854 | 1.4721 | 0.0457 | clean |
| Corncob | genus | Tardiphaga | -4.9594 | -8.3673 | -1.5516 | 1.1419 | 0.0330 | clean |
| Corncob | family | Xanthobacteraceae | -5.4684 | -9.5934 | -1.3435 | 1.3822 | 0.0447 | clean |

| B. Great tit (*Parus major*) | | | | | | | | |
| --- | --- | --- | --- | --- | --- | --- | --- | --- |
| **DA tool** | **Level** | **Taxon** | **Log2FC** | **Lower** | **Upper** | **SE** | **Adj p** | **Elevated in** |
| DESeq2 | phylum | Acidobacteria | -6.9252 | -11.1738 | -2.6765 | 2.1677 | 0.0090 | clean |
| DESeq2 | class | Acidobacteria_Gp16 | -9.2777 | -13.5167 | -5.0386 | 2.1628 | 0.0002 | clean |
| DESeq2 | genus | Actinoallomurus | -5.2178 | -9.1858 | -1.2499 | 2.0245 | 0.0362 | clean |
| Corncob | genus | Actinoallomurus | -6.2104 | -10.7094 | -1.7114 | 1.5199 | 0.0377 | clean |
| DESeq2 | genus | Actinomadura | -24.9834 | -30.0646 | -19.9021 | 2.5925 | 0.0000 | clean |
| Corncob | genus | Actinomadura | -8.6056 | -15.1232 | -2.0880 | 2.2018 | 0.0482 | clean |
| DESeq2 | genus | Actinomycetospora | -6.1850 | -10.1759 | -2.1941 | 2.0362 | 0.0113 | clean |
| Corncob | genus | Actinoplanes | -7.6043 | -12.3973 | -2.8113 | 1.6192 | 0.0142 | clean |
| Corncob | genus | Agromyces | -7.3354 | -12.1702 | -2.5007 | 1.6333 | 0.0214 | clean |
| DESeq2 | genus | Amnibacterium | -6.1348 | -10.5049 | -1.7646 | 2.2297 | 0.0259 | clean |
| Corncob | genus | Anaerovorax | -7.7426 | -13.3720 | -2.1132 | 1.9017 | 0.0377 | clean |
| DESeq2 | genus | Aquabacterium | 26.7481 | 20.9960 | 32.5001 | 2.9347 | 0.0000 | polluted |
| Corncob | genus | Aquicella | 6.8469 | 1.8366 | 11.8571 | 1.6926 | 0.0379 | polluted |
| DESeq2 | genus | Aquisphaera | -24.2870 | -29.5973 | -18.9766 | 2.7094 | 0.0000 | clean |
| LinDA | genus | Arthrobacter | -5.3353 | -8.5015 | -2.1692 | 1.5481 | 0.0480 | clean |
| DESeq2 | genus | Arthrobacter | -3.7967 | -6.7712 | -0.8222 | 1.5176 | 0.0434 | clean |
| Corncob | genus | Arthrobacter | -6.6059 | -9.6752 | -3.5367 | 1.0369 | 0.0005 | clean |
| Corncob | family | Bacillaceae_1 | -3.8566 | -6.4155 | -1.2977 | 0.8645 | 0.0216 | clean |
| Corncob | order | Bacillales | -4.5368 | -6.3665 | -2.7071 | 0.6181 | 0.0000 | clean |
| DESeq2 | class | Bacilli | 3.8781 | 2.1576 | 5.5986 | 0.8778 | 0.0002 | polluted |
| DESeq2 | order | Bacteroidales | 10.6762 | 5.7758 | 15.5765 | 2.5002 | 0.0003 | polluted |
| DESeq2 | phylum | Bacteroidetes | 5.5187 | 2.2999 | 8.7376 | 1.6422 | 0.0090 | polluted |
| DESeq2 | class | Bacteroidia | 10.7957 | 5.7604 | 15.8310 | 2.569 | 0.0002 | polluted |
| DESeq2 | class | Betaproteobacteria | 3.3303 | 0.8345 | 5.8262 | 1.2734 | 0.0267 | polluted |
| Corncob | genus | Bosea | -7.7918 | -11.3517 | -4.2319 | 1.2026 | 0.0005 | clean |
| Corncob | family | Brevibacteriaceae | -10.0384 | -13.8297 | -6.2472 | 1.2808 | 0.0000 | clean |
| Corncob | genus | Brevibacterium | -10.7314 | -14.5338 | -6.9291 | 1.2845 | 0.0000 | clean |
| DESeq2 | family | Brucellaceae | -6.1766 | -10.2042 | -2.1490 | 2.0549 | 0.0217 | clean |
| DESeq2 | family | Burkholderiales_incertae_sedis | 28.4683 | 22.7161 | 34.2205 | 2.9348 | 0.0000 | polluted |
| DESeq2 | family | Carnobacteriaceae | 7.7095 | 4.7109 | 10.7081 | 1.5299 | 0.0000 | polluted |
| LinDA | genus | Catellicoccus | 8.0835 | 3.3677 | 12.7992 | 2.3057 | 0.0480 | polluted |
| DESeq2 | genus | Catellicoccus | 15.0120 | 9.9566 | 20.0674 | 2.5793 | 0.0000 | polluted |
| ANCOM-BC2 | genus | Catellicoccus | 5.4542 | 2.4186 | 8.4898 | 1.5488 | 0.0234 | polluted |
| Corncob | family | Caulobacteraceae | 4.6680 | 1.5701 | 7.7659 | 1.0465 | 0.0216 | polluted |
| Corncob | order | Caulobacterales | 4.8754 | 1.7799 | 7.9708 | 1.0457 | 0.0222 | polluted |
| DESeq2 | phylum | Chloroflexi | -6.3934 | -11.5518 | -1.2349 | 2.6319 | 0.0492 | clean |
| DESeq2 | genus | Clostridium_XVIII | 7.2331 | 2.7299 | 11.7363 | 2.2975 | 0.0081 | polluted |
| DESeq2 | genus | Cohnella | -6.0895 | -10.9241 | -1.2549 | 2.4666 | 0.0462 | clean |
| DESeq2 | genus | Conexibacter | -4.6356 | -8.0218 | -1.2495 | 1.7276 | 0.0284 | clean |
| DESeq2 | family | Corynebacterineae_incertae_sedis | -8.2158 | -12.2567 | -4.1748 | 2.0617 | 0.0009 | clean |
| DESeq2 | class | Deltaproteobacteria | -4.9053 | -7.9666 | -1.844 | 1.5619 | 0.0071 | clean |
| DESeq2 | order | Enterobacteriales | 5.3886 | 2.2352 | 8.5420 | 1.6089 | 0.0050 | polluted |
| DESeq2 | family | Enterococcaceae | 3.1669 | 1.0497 | 5.2841 | 1.0802 | 0.0253 | polluted |
| DESeq2 | genus | Enterococcus | 6.1856 | 3.4738 | 8.8973 | 1.3835 | 0.0001 | polluted |
| DESeq2 | genus | Erysipelothrix | -23.6505 | -29.4033 | -17.8978 | 2.9351 | 0.0000 | clean |
| DESeq2 | order | Erysipelotrichales | 5.9856 | 2.6626 | 9.3087 | 1.6954 | 0.0037 | polluted |
| DESeq2 | class | Erysipelotrichia | 6.3607 | 3.2104 | 9.5110 | 1.6073 | 0.0004 | polluted |
| DESeq2 | genus | Escherichia/Shigella | 15.9850 | 10.8833 | 21.0868 | 2.6029 | 0.0000 | polluted |
| DESeq2 | phylum | Firmicutes | 2.1469 | 0.7801 | 3.5138 | 0.6974 | 0.0090 | polluted |
| DESeq2 | genus | Gordonibacter | 7.9154 | 2.1866 | 13.6442 | 2.9229 | 0.0284 | polluted |
| DESeq2 | genus | Gp16 | -6.0447 | -10.1455 | -1.9440 | 2.0922 | 0.0175 | clean |
| Corncob | genus | Janibacter | -7.0081 | -11.8318 | -2.1844 | 1.6295 | 0.0269 | clean |
| DESeq2 | genus | Kaistia | -24.6606 | -29.6715 | -19.6498 | 2.5566 | 0.0000 | clean |
| DESeq2 | family | Lactobacillaceae | 8.8125 | 5.1069 | 12.5182 | 1.8906 | 0.0001 | polluted |
| Corncob | family | Lactobacillaceae | 7.8969 | 3.9083 | 11.8855 | 1.3474 | 0.0012 | polluted |
| DESeq2 | order | Lactobacillales | 5.1288 | 3.0767 | 7.1809 | 1.0470 | 0.0000 | polluted |
| DESeq2 | genus | Lactobacillus | 7.2458 | 3.7215 | 10.7702 | 1.7981 | 0.0004 | polluted |
| Corncob | genus | Lactobacillus | 7.9140 | 3.9678 | 11.8602 | 1.3331 | 0.0015 | polluted |
| DESeq2 | genus | Lactococcus | 22.8636 | 17.1087 | 28.6185 | 2.9362 | 0.0000 | polluted |
| Corncob | genus | Leuconostoc | -6.9111 | -10.9654 | -2.8567 | 1.3696 | 0.0064 | clean |
| Corncob | family | Leuconostocaceae | -7.0561 | -11.1396 | -2.9725 | 1.3795 | 0.0056 | clean |
| Corncob | genus | Lysinibacillus | -6.5700 | -9.8997 | -3.2404 | 1.1248 | 0.0015 | clean |
| LinDA | family | Micrococcaceae | -5.3481 | -8.2852 | -2.4111 | 1.4361 | 0.0438 | clean |
| Corncob | family | Micrococcaceae | -6.6785 | -9.4946 | -3.8624 | 0.9513 | 0.0000 | clean |
| LinDA | family | Mycobacteriaceae | -4.9981 | -7.5103 | -2.4859 | 1.2283 | 0.0345 | clean |
| DESeq2 | family | Mycobacteriaceae | -6.3186 | -9.1471 | -3.4901 | 1.4431 | 0.0002 | clean |
| LinDA | genus | Mycobacterium | -4.7978 | -7.2971 | -2.2985 | 1.2220 | 0.0314 | clean |
| DESeq2 | genus | Mycobacterium | -6.7193 | -9.5937 | -3.8448 | 1.4666 | 0.0000 | clean |
| DESeq2 | order | Myxococcales | -7.1982 | -11.4216 | -2.9748 | 2.1548 | 0.0050 | clean |
| DESeq2 | genus | Nakamurella | -8.4524 | -12.3223 | -4.5824 | 1.9744 | 0.0001 | clean |
| DESeq2 | family | Nakamurellaceae | -7.4617 | -11.1910 | -3.7324 | 1.9027 | 0.0010 | clean |
| DESeq2 | genus | Nocardia | -9.2684 | -13.5739 | -4.9628 | 2.1967 | 0.0002 | clean |
| Corncob | genus | Nocardia | -9.7234 | -14.7924 | -4.6544 | 1.7124 | 0.0021 | clean |
| DESeq2 | family | Nocardiaceae | -4.6790 | -7.5900 | -1.7680 | 1.4852 | 0.0147 | clean |
| Corncob | family | Nocardiaceae | -5.7582 | -9.0007 | -2.5157 | 1.0954 | 0.0045 | clean |
| DESeq2 | genus | Ochrobactrum | -23.1740 | -28.1252 | -18.2228 | 2.5261 | 0.0000 | clean |
| DESeq2 | genus | Paenibacillus | -6.3712 | -10.0528 | -2.6895 | 1.8784 | 0.0038 | clean |
| Corncob | genus | Paenibacillus | -6.4276 | -11.0571 | -1.7981 | 1.5639 | 0.0377 | clean |
| Corncob | family | Planococcaceae | -6.6791 | -9.4691 | -3.8891 | 0.9425 | 0.0000 | clean |
| DESeq2 | genus | Pseudochrobactrum | -6.7974 | -11.9560 | -1.6387 | 2.6320 | 0.0362 | clean |
| Corncob | genus | Pseudochrobactrum | -8.5142 | -13.4142 | -3.6141 | 1.6553 | 0.0055 | clean |
| Corncob | genus | Pseudomonas | -2.9507 | -4.9421 | -0.9593 | 0.6727 | 0.0253 | clean |
| DESeq2 | family | Pseudonocardiaceae | -6.3226 | -9.9249 | -2.7202 | 1.8380 | 0.0058 | clean |
| LinDA | genus | Rhodococcus | -4.6006 | -7.2015 | -1.9997 | 1.2717 | 0.0458 | clean |
| DESeq2 | genus | Rhodococcus | -6.3523 | -9.6388 | -3.0658 | 1.6768 | 0.0009 | clean |
| Corncob | genus | Rhodococcus | -6.8854 | -10.6198 | -3.1511 | 1.2615 | 0.0028 | clean |
| DESeq2 | family | Roseiarcaceae | -5.5997 | -9.6088 | -1.5907 | 2.0454 | 0.0428 | clean |
| DESeq2 | genus | Roseiarcus | -6.4333 | -10.2878 | -2.5788 | 1.9666 | 0.0056 | clean |
| LinDA | genus | Serratia | 3.5582 | 1.6758 | 5.4407 | 0.9204 | 0.0314 | polluted |
| DESeq2 | genus | Serratia | 23.5468 | 18.5171 | 28.5765 | 2.5662 | 0.0000 | polluted |
| ANCOM-BC2 | genus | Serratia | 2.3010 | 1.1188 | 3.4831 | 0.6032 | 0.0112 | polluted |
| LinDA | genus | Sodalis | 3.2099 | 1.7543 | 4.6655 | 0.7117 | 0.0162 | polluted |
| ANCOM-BC2 | genus | Sodalis | 2.0589 | 1.1665 | 2.9513 | 0.4553 | 0.0010 | polluted |
| DESeq2 | order | Sphingomonadales | 6.2311 | 3.3613 | 9.1009 | 1.4642 | 0.0003 | polluted |
| DESeq2 | genus | Sphingomonas | 3.9612 | 1.0742 | 6.8482 | 1.4730 | 0.0284 | polluted |
| Corncob | genus | Sporosarcina | -8.2716 | -12.7786 | -3.7647 | 1.5225 | 0.0028 | clean |
| DESeq2 | family | Staphylococcaceae | -11.0303 | -15.3140 | -6.7467 | 2.1855 | 0.0000 | clean |
| DESeq2 | genus | Staphylococcus | -23.9756 | -29.7269 | -18.2243 | 2.9343 | 0.0000 | clean |
| DESeq2 | family | Streptococcaceae | 29.6400 | 23.8881 | 35.3919 | 2.9346 | 0.0000 | polluted |
| Corncob | genus | Streptomyces | -7.8018 | -13.1584 | -2.4452 | 1.8096 | 0.0269 | clean |
| DESeq2 | class | Thermomicrobia | -6.0798 | -10.2971 | -1.8625 | 2.1517 | 0.0165 | clean |
| Corncob | family | Thermomonosporaceae | -6.1297 | -10.2299 | -2.0295 | 1.3851 | 0.0216 | clean |
| DESeq2 | genus | Tomitella | -8.6319 | -12.8687 | -4.3951 | 2.1616 | 0.0004 | clean |
| Corncob | genus | Tumebacillus | -9.1131 | -14.0227 | -4.2035 | 1.6586 | 0.0028 | clean |
| Corncob | family | Veillonellaceae | -10.4624 | -16.0157 | -4.9091 | 1.8760 | 0.0022 | clean |
| DESeq2 | genus | Williamsia | -23.8019 | -29.5539 | -18.0499 | 2.9347 | 0.0000 | clean |

| C. Pied flycatcher (*Ficedula hypoleuca*) | | | | | | | | |
| --- | --- | --- | --- | --- | --- | --- | --- | --- |
| **DA tool** | **Level** | **Taxon** | **Log2FC** | **Lower** | **Upper** | **SE** | **Adj p** | **Elevated in** |
| DESeq2 | class | Acidobacteria_Gp16 | 5.9346 | 2.5740 | 9.2952 | 1.7146 | 0.0048 | polluted |
| DESeq2 | genus | Actinomadura | -4.5616 | -7.8473 | -1.2758 | 1.6764 | 0.0288 | clean |
| DESeq2 | genus | Aeromicrobium | 7.1989 | 3.6333 | 10.7644 | 1.8192 | 0.0006 | polluted |
| Corncob | genus | Akkermansia | -8.5586 | -12.1925 | -4.9247 | 1.2254 | 0.0000 | clean |
| DESeq2 | class | Alphaproteobacteria | 2.0043 | 0.7341 | 3.2745 | 0.6481 | 0.0107 | polluted |
| DESeq2 | genus | Anaerotruncus | -24.4877 | -29.3378 | -19.6375 | 2.4746 | 0.0000 | clean |
| ANCOM-BC2 | genus | Anaerotruncus | -2.3492 | -3.6843 | -1.0142 | 0.6812 | 0.0226 | clean |
| LinDA | genus | Anaerovorax | -3.9712 | -6.0038 | -1.9385 | 0.9923 | 0.0262 | clean |
| DESeq2 | genus | Anaerovorax | -24.8910 | -29.3098 | -20.4722 | 2.2545 | 0.0000 | clean |
| ANCOM-BC2 | genus | Anaerovorax | -2.4169 | -3.7540 | -1.0798 | 0.6822 | 0.0206 | clean |
| Corncob | family | Bacillaceae_1 | 3.3596 | 0.7489 | 5.9703 | 0.8804 | 0.0469 | polluted |
| DESeq2 | order | Bacillales | 3.1392 | 1.3364 | 4.9421 | 0.9198 | 0.0045 | clean |
| Corncob | order | Bacillales | 3.1390 | 1.2369 | 5.0410 | 0.6414 | 0.0076 | polluted |
| DESeq2 | class | Bacilli | 2.5488 | 1.0639 | 4.0337 | 0.7576 | 0.0052 | polluted |
| DESeq2 | genus | Bacillus | 5.3225 | 2.7560 | 7.8891 | 1.3095 | 0.0004 | polluted |
| LinDA | family | Bacteroidaceae | -6.6754 | -9.8838 | -3.4670 | 1.5663 | 0.0064 | clean |
| ANCOM-BC2 | family | Bacteroidaceae | -4.2368 | -6.5100 | -1.9635 | 1.1598 | 0.0054 | clean |
| LinDA | order | Bacteroidales | -6.2897 | -9.6980 | -2.8815 | 1.6639 | 0.0362 | clean |
| ANCOM-BC2 | order | Bacteroidales | -4.3044 | -7.0229 | -1.5858 | 1.3870 | 0.0459 | clean |
| ANCOM-BC2 | genus | Bacteroides | -3.5196 | -5.8056 | -1.2337 | 1.1663 | 0.0499 | clean |
| DESeq2 | phylum | Bacteroidetes | -4.3145 | -6.7761 | -1.8529 | 1.2559 | 0.0024 | clean |
| LinDA | class | Bacteroidia | -5.8332 | -9.1807 | -2.4857 | 1.6342 | 0.0368 | clean |
| Corncob | family | Bdellovibrionaceae | -9.6751 | -16.6035 | -2.7466 | 2.3364 | 0.0295 | clean |
| DESeq2 | genus | Blastococcus | 4.8097 | 1.2437 | 8.3758 | 1.8194 | 0.0321 | polluted |
| Corncob | genus | Blautia | 7.9846 | 2.3552 | 13.6141 | 1.8983 | 0.0310 | polluted |
| Corncob | family | Brucellaceae | 10.2731 | 4.9978 | 15.5484 | 1.7789 | 0.0009 | polluted |
| Corncob | family | Carnobacteriaceae | -3.7038 | -6.3896 | -1.0180 | 0.9057 | 0.0310 | clean |
| Corncob | genus | Carnobacterium | -4.1879 | -6.9451 | -1.4307 | 0.9298 | 0.0180 | clean |
| LinDA | genus | Catabacter | -3.1473 | -4.7103 | -1.5843 | 0.7630 | 0.0262 | clean |
| DESeq2 | genus | Catabacter | -5.7738 | -10.0048 | -1.5428 | 2.1587 | 0.0311 | clean |
| ANCOM-BC2 | genus | Catabacter | -1.8458 | -2.8240 | -0.8677 | 0.4991 | 0.0180 | clean |
| LinDA | family | Catabacteriaceae | -3.3715 | -4.8701 | -1.8728 | 0.7316 | 0.0051 | clean |
| ANCOM-BC2 | family | Catabacteriaceae | -1.9615 | -2.8685 | -1.0545 | 0.4628 | 0.0014 | clean |
| Corncob | family | Cellulomonadaceae | -4.0939 | -6.4218 | -1.7660 | 0.7850 | 0.0035 | clean |
| Corncob | genus | Cellulomonas | -4.0594 | -6.3811 | -1.7377 | 0.7829 | 0.0050 | clean |
| DESeq2 | order | Chlamydiales | -3.5766 | -5.9939 | -1.1593 | 1.2333 | 0.0196 | polluted |
| DESeq2 | class | Chlamydiia | -3.1985 | -5.5272 | -0.8697 | 1.1881 | 0.0240 | clean |
| DESeq2 | phylum | Chloroflexi | 4.9740 | 1.1123 | 8.8358 | 1.9703 | 0.0371 | polluted |
| LinDA | family | Christensenellaceae | -3.1701 | -4.5662 | -1.7739 | 0.6816 | 0.0051 | clean |
| ANCOM-BC2 | family | Christensenellaceae | -1.8219 | -2.5709 | -1.0729 | 0.3822 | 0.0002 | clean |
| LinDA | family | Clostridiales_Incertae_Sedis_XIII | -4.1953 | -6.1324 | -2.2583 | 0.9456 | 0.0054 | clean |
| DESeq2 | family | Clostridiales_Incertae_Sedis_XIII | -7.0812 | -11.0863 | -3.0760 | 2.0434 | 0.0056 | clean |
| ANCOM-BC2 | family | Clostridiales_Incertae_Sedis_XIII | -2.5326 | -3.8373 | -1.2278 | 0.6657 | 0.0045 | clean |
| Corncob | genus | Clostridium_IV | -4.8262 | -8.3856 | -1.2667 | 1.2003 | 0.0411 | clean |
| DESeq2 | genus | Clostridium_XlVb | -5.7043 | -9.8839 | -1.5246 | 2.1325 | 0.0311 | clean |
| ANCOM-BC2 | genus | Clostridium_XlVb | -2.2422 | -3.6060 | -0.8784 | 0.6958 | 0.0367 | clean |
| ANCOM-BC2 | genus | Clostridium_XVIII | -4.1035 | -6.6065 | -1.6005 | 1.2770 | 0.0367 | clean |
| DESeq2 | genus | Coprococcus | -23.8321 | -28.7685 | -18.8957 | 2.5186 | 0.0000 | clean |
| ANCOM-BC2 | genus | Coprococcus | -1.9851 | -3.2789 | -0.6914 | 0.6601 | 0.0499 | clean |
| LinDA | order | Coriobacteriales | -5.6235 | -9.1392 | -2.1077 | 1.7163 | 0.0449 | clean |
| DESeq2 | order | Cytophagales | -28.6367 | -34.3856 | -22.8877 | 2.9331 | 0.0000 | polluted |
| DESeq2 | class | Cytophagia | -28.0579 | -33.8072 | -22.3085 | 2.9334 | 0.0000 | clean |
| DESeq2 | genus | Devosia | 6.5077 | 3.9193 | 9.0961 | 1.3206 | 0.0000 | polluted |
| Corncob | genus | Devosia | 7.8061 | 5.4238 | 10.1885 | 0.8034 | 0.0000 | polluted |
| DESeq2 | family | Enterobacteriaceae | 3.2404 | 1.3164 | 5.1643 | 0.9816 | 0.0084 | polluted |
| Corncob | family | Enterobacteriaceae | 2.5878 | 0.8392 | 4.3364 | 0.5896 | 0.0208 | polluted |
| DESeq2 | order | Enterobacteriales | 3.7931 | 1.9455 | 5.6408 | 0.9427 | 0.0006 | polluted |
| Corncob | order | Enterobacteriales | 2.7380 | 0.9648 | 4.5112 | 0.5979 | 0.0132 | polluted |
| DESeq2 | genus | Escherichia/Shigella | 11.2625 | 6.7865 | 15.7386 | 2.2837 | 0.0000 | polluted |
| Corncob | genus | Escherichia/Shigella | 9.5195 | 4.1909 | 14.8482 | 1.7969 | 0.0040 | polluted |
| LinDA | family | Eubacteriaceae | -4.6937 | -7.1554 | -2.2320 | 1.2018 | 0.0114 | clean |
| ANCOM-BC2 | family | Eubacteriaceae | -2.9289 | -4.8043 | -1.0535 | 0.9569 | 0.0347 | clean |
| LinDA | genus | Eubacterium | -5.8411 | -8.3114 | -3.3709 | 1.2059 | 0.0103 | clean |
| DESeq2 | genus | Eubacterium | -8.9181 | -12.1818 | -5.6544 | 1.6652 | 0.0000 | clean |
| ANCOM-BC2 | genus | Eubacterium | -3.5190 | -5.1141 | -1.9239 | 0.8138 | 0.0037 | clean |
| Corncob | genus | Eubacterium | -10.5092 | -13.6117 | -7.4068 | 1.0462 | 0.0000 | clean |
| DESeq2 | phylum | Fusobacteria | 12.0135 | 6.5534 | 17.4736 | 2.7858 | 0.0001 | polluted |
| Corncob | phylum | Fusobacteria | 13.4029 | 8.5047 | 18.3011 | 1.6517 | 0.0000 | polluted |
| DESeq2 | family | Fusobacteriaceae | 9.2600 | 4.4391 | 14.0809 | 2.4596 | 0.0027 | polluted |
| Corncob | family | Fusobacteriaceae | 12.8801 | 7.8990 | 17.8612 | 1.6797 | 0.0000 | polluted |
| DESeq2 | order | Fusobacteriales | 10.4812 | 5.0290 | 15.9335 | 2.7818 | 0.0014 | polluted |
| Corncob | order | Fusobacteriales | 13.3939 | 8.5063 | 18.2814 | 1.6481 | 0.0000 | polluted |
| DESeq2 | class | Fusobacteriia | 11.1907 | 6.2579 | 16.1236 | 2.5167 | 0.0001 | polluted |
| Corncob | class | Fusobacteriia | 13.4150 | 8.5188 | 18.3112 | 1.6511 | 0.0000 | polluted |
| DESeq2 | genus | Fusobacterium | 9.9433 | 4.6206 | 15.2659 | 2.7156 | 0.0018 | polluted |
| Corncob | genus | Fusobacterium | 12.8866 | 7.8783 | 17.8949 | 1.6889 | 0.0000 | polluted |
| DESeq2 | class | Gammaproteobacteria | 1.9545 | 0.6084 | 3.3006 | 0.6868 | 0.0199 | polluted |
| ANCOM-BC2 | genus | Gordonibacter | -3.9660 | -6.1729 | -1.7590 | 1.1260 | 0.0206 | clean |
| DESeq2 | genus | Gp16 | 5.7720 | 2.2304 | 9.3135 | 1.8069 | 0.0075 | polluted |
| DESeq2 | genus | Hafnia | 4.8410 | 1.5730 | 8.1089 | 1.6673 | 0.0182 | polluted |
| DESeq2 | genus | Hespellia | -22.5720 | -28.3267 | -16.8173 | 2.9361 | 0.0000 | clean |
| DESeq2 | family | Hyphomicrobiaceae | 5.8203 | 3.5631 | 8.0775 | 1.1516 | 0.0000 | polluted |
| Corncob | family | Hyphomicrobiaceae | 7.1114 | 4.8684 | 9.3544 | 0.7564 | 0.0000 | polluted |
| DESeq2 | genus | Ilumatobacter | 4.8079 | 1.4732 | 8.1425 | 1.7013 | 0.0224 | polluted |
| ANCOM-BC2 | genus | Intestinimonas | -1.7357 | -2.8277 | -0.6437 | 0.5571 | 0.0443 | clean |
| DESeq2 | genus | Janibacter | 6.1007 | 2.2883 | 9.9131 | 1.9451 | 0.0087 | polluted |
| DESeq2 | genus | Kaistia | 22.3641 | 16.6755 | 28.0526 | 2.9023 | 0.0000 | polluted |
| Corncob | genus | Knoellia | 9.5977 | 4.4109 | 14.7844 | 1.7490 | 0.0032 | polluted |
| DESeq2 | class | Ktedonobacteria | 5.7778 | 1.6324 | 9.9231 | 2.1149 | 0.024 | polluted |
| LinDA | family | Lachnospiraceae | -3.9237 | -6.4147 | -1.4328 | 1.2160 | 0.0446 | clean |
| DESeq2 | family | Lachnospiraceae | -2.6040 | -4.3922 | -0.8158 | 0.9123 | 0.0319 | clean |
| Corncob | genus | Leuconostoc | 6.5297 | 3.9022 | 9.1571 | 0.8860 | 0.0000 | polluted |
| DESeq2 | family | Leuconostocaceae | 4.4483 | 1.8630 | 7.0337 | 1.3191 | 0.0072 | polluted |
| Corncob | family | Leuconostocaceae | 7.5985 | 5.1839 | 10.0131 | 0.8142 | 0.0000 | polluted |
| Corncob | genus | Listeria | 8.2727 | 3.3485 | 13.1969 | 1.6605 | 0.0080 | polluted |
| Corncob | family | Listeriaceae | 12.4205 | 7.7673 | 17.0737 | 1.5691 | 0.0000 | polluted |
| Corncob | genus | Micromonospora | 6.5141 | 2.0863 | 10.9419 | 1.4931 | 0.0228 | polluted |
| Corncob | family | Micromonosporaceae | 4.7724 | 1.4597 | 8.0850 | 1.1171 | 0.0240 | polluted |
| ANCOM-BC2 | genus | Mucinivorans | -1.0746 | -1.6454 | -0.5038 | 0.2912 | 0.0180 | clean |
| DESeq2 | genus | Nitrososphaera | 24.8341 | 19.9190 | 29.7491 | 2.5077 | 0.0000 | polluted |
| DESeq2 | family | Nitrososphaeraceae | 24.3502 | 19.9154 | 28.7851 | 2.2627 | 0.0000 | polluted |
| DESeq2 | genus | Nocardia | 4.8215 | 1.8645 | 7.7785 | 1.5087 | 0.0075 | polluted |
| Corncob | genus | Nocardia | 4.2765 | 1.4903 | 7.0627 | 0.9395 | 0.0170 | polluted |
| Corncob | family | Nocardiaceae | 3.4866 | 1.0608 | 5.9124 | 0.8180 | 0.0240 | polluted |
| Corncob | genus | Obesumbacterium | 6.1507 | 2.8349 | 9.4665 | 1.1181 | 0.0032 | polluted |
| DESeq2 | family | Paenibacillaceae_1 | 3.3149 | 1.0769 | 5.5529 | 1.1418 | 0.0296 | polluted |
| Corncob | family | Paenibacillaceae_1 | 5.3432 | 2.8201 | 7.8663 | 0.8508 | 0.0002 | polluted |
| DESeq2 | genus | Paenibacillus | 3.3981 | 0.9806 | 5.8155 | 1.2334 | 0.0269 | polluted |
| Corncob | genus | Paenibacillus | 5.0210 | 2.5438 | 7.4982 | 0.8353 | 0.0009 | polluted |
| DESeq2 | genus | Parabacteroides | -9.3526 | -13.4303 | -5.2748 | 2.0805 | 0.0001 | clean |
| DESeq2 | family | Parachlamydiaceae | -5.7333 | -8.8668 | -2.5998 | 1.5987 | 0.0040 | clean |
| Corncob | family | Parachlamydiaceae | -6.0248 | -8.5398 | -3.5097 | 0.8481 | 0.0000 | clean |
| DESeq2 | genus | Paracoccus | 8.1285 | 4.0616 | 12.1954 | 2.0749 | 0.0007 | polluted |
| Corncob | genus | Paracoccus | 10.4550 | 7.1421 | 13.7678 | 1.1171 | 0.0000 | polluted |
| DESeq2 | genus | Phycicoccus | 5.3066 | 1.3561 | 9.2572 | 2.0156 | 0.0322 | polluted |
| DESeq2 | phylum | Proteobacteria | 2.7387 | 1.5105 | 3.9669 | 0.6266 | 0.0001 | polluted |
| Corncob | phylum | Proteobacteria | 1.7652 | 0.5813 | 2.9492 | 0.3992 | 0.0174 | polluted |
| DESeq2 | genus | Providencia | -6.9159 | -12.0416 | -1.7902 | 2.6151 | 0.0321 | clean |
| DESeq2 | genus | Pseudolabrys | 4.7871 | 2.1693 | 7.4049 | 1.3356 | 0.0022 | polluted |
| ANCOM-BC2 | family | Pseudomonadaceae | -3.1807 | -4.8522 | -1.5092 | 0.8528 | 0.0048 | clean |
| Corncob | family | Pseudomonadaceae | -2.9418 | -4.6832 | -1.2005 | 0.5872 | 0.0054 | clean |
| ANCOM-BC2 | order | Pseudomonadales | -3.0737 | -4.8720 | -1.2755 | 0.9175 | 0.0388 | clean |
| Corncob | order | Pseudomonadales | -2.4136 | -4.1002 | -0.7270 | 0.5687 | 0.0239 | clean |
| Corncob | genus | Pseudomonas | -2.6415 | -4.5427 | -0.7404 | 0.6411 | 0.0359 | clean |
| LinDA | family | Rhodobacteraceae | 5.0124 | 1.9499 | 8.0748 | 1.4950 | 0.0363 | polluted |
| DESeq2 | family | Rhodobacteraceae | 8.1401 | 5.0261 | 11.2541 | 1.5888 | 0.0000 | polluted |
| ANCOM-BC2 | family | Rhodobacteraceae | 3.2714 | 1.2164 | 5.3263 | 1.0484 | 0.0325 | polluted |
| Corncob | family | Rhodobacteraceae | 10.8004 | 7.7074 | 13.8933 | 1.0430 | 0.0000 | polluted |
| LinDA | order | Rhodobacterales | 4.8601 | 1.8504 | 7.8697 | 1.4693 | 0.0449 | polluted |
| DESeq2 | order | Rhodobacterales | 8.8276 | 5.8193 | 11.8360 | 1.5349 | 0.0000 | polluted |
| ANCOM-BC2 | order | Rhodobacterales | 3.2863 | 1.1230 | 5.4496 | 1.1037 | 0.0465 | polluted |
| Corncob | order | Rhodobacterales | 10.9217 | 7.7464 | 14.0969 | 1.0707 | 0.0000 | polluted |
| LinDA | genus | Rhodococcus | 4.3132 | 2.0970 | 6.5294 | 1.0819 | 0.0262 | polluted |
| DESeq2 | genus | Rhodococcus | 4.6827 | 1.8952 | 7.4702 | 1.4222 | 0.0057 | polluted |
| DESeq2 | order | Rickettsiales | 22.1882 | 16.4263 | 27.9500 | 2.9397 | 0.0000 | clean |
| DESeq2 | genus | Rikenella | -23.6752 | -28.9295 | -18.4209 | 2.6808 | 0.0000 | clean |
| ANCOM-BC2 | genus | Rikenella | -1.9551 | -3.2321 | -0.6781 | 0.6515 | 0.0499 | clean |
| DESeq2 | family | Rikenellaceae | -8.3128 | -11.8434 | -4.7823 | 1.8013 | 0.0001 | clean |
| LinDA | family | Roseiarcaceae | -3.0413 | -4.8827 | -1.2000 | 0.8989 | 0.0363 | clean |
| Corncob | family | Roseiarcaceae | -3.7705 | -6.5938 | -0.9472 | 0.9520 | 0.0366 | clean |
| Corncob | genus | Roseiarcus | -3.8450 | -6.6783 | -1.0116 | 0.9554 | 0.0411 | clean |
| LinDA | family | Ruminococcaceae | -6.7514 | -10.0553 | -3.4475 | 1.6129 | 0.0064 | clean |
| ANCOM-BC2 | family | Ruminococcaceae | -4.4373 | -6.6714 | -2.2031 | 1.1399 | 0.0042 | clean |
| DESeq2 | genus | Sanguibacter | 5.7275 | 2.5499 | 8.9051 | 1.6212 | 0.0026 | polluted |
| Corncob | genus | Sanguibacter | 5.8251 | 2.1665 | 9.4837 | 1.2337 | 0.0126 | polluted |
| DESeq2 | family | Sanguibacteraceae | 5.5693 | 2.5311 | 8.6074 | 1.5501 | 0.0040 | polluted |
| Corncob | family | Sanguibacteraceae | 5.9521 | 2.2003 | 9.7039 | 1.2651 | 0.0106 | polluted |
| DESeq2 | genus | Serratia | 9.5452 | 4.0465 | 15.0439 | 2.8055 | 0.0040 | polluted |
| DESeq2 | genus | Subdoligranulum | -7.6373 | -11.6919 | -3.5826 | 2.0687 | 0.0016 | clean |
| ANCOM-BC2 | genus | Subdoligranulum | -2.9952 | -4.8292 | -1.1612 | 0.9357 | 0.0367 | clean |
| DESeq2 | family | Synergistaceae | 22.8176 | 17.0653 | 28.5698 | 2.9348 | 0.0000 | polluted |
| Corncob | genus | Tardiphaga | 5.4198 | 1.9157 | 8.9239 | 1.1816 | 0.0165 | polluted |
| Corncob | genus | Terrabacter | 8.4098 | 3.2204 | 13.5993 | 1.7499 | 0.0115 | polluted |
| DESeq2 | phylum | Thaumarchaeota | 23.3619 | 18.1279 | 28.5960 | 2.6704 | 0.0000 | polluted |
| Corncob | genus | Thermoactinomyces | 9.1449 | 4.1595 | 14.1302 | 1.6811 | 0.0033 | polluted |
| Corncob | family | Thermoactinomycetaceae_1 | 9.1715 | 4.1451 | 14.1979 | 1.6950 | 0.0023 | polluted |
| DESeq2 | genus | Thermogemmatispora | 22.9070 | 17.6936 | 28.1204 | 2.6599 | 0.0000 | polluted |
| DESeq2 | order | Thermogemmatisporales | 7.5285 | 2.6709 | 12.3862 | 2.4784 | 0.0143 | clean |
| Corncob | order | Thermogemmatisporales | 8.6552 | 3.4665 | 13.8439 | 1.7497 | 0.0076 | polluted |
| DESeq2 | genus | Tomitella | 22.3286 | 16.5738 | 28.0834 | 2.9361 | 0.0000 | polluted |
| DESeq2 | genus | Turicibacter | 11.3617 | 7.1191 | 15.6042 | 2.1646 | 0.0000 | polluted |
| Corncob | genus | Turicibacter | 9.1573 | 3.0996 | 15.2149 | 2.0427 | 0.0181 | polluted |
| Corncob | family | Verrucomicrobiaceae | -4.9977 | -8.6636 | -1.3319 | 1.2362 | 0.0324 | clean |
| Corncob | genus | Weissella | 10.0488 | 4.5091 | 15.5885 | 1.8680 | 0.0035 | polluted |
| Corncob | genus | Yersinia | -8.6271 | -13.9768 | -3.2773 | 1.8040 | 0.0115 | clean |

# References

Apprill A, McNally S, Parsons R, Weber L. Minor revision to V4 region SSU rRNA 806R gene primer greatly increases detection of SAR11 bacterioplankton. Aquat Microb Ecol 2015; 75: 129-137.

Caporaso JG, Lauber CL, Walters WA, Berg-Lyons D, Lozupone CA, Turnbaugh PJ, et al. Global patterns of 16S rRNA diversity at a depth of millions of sequences per sample. P Natl Acad Sci USA 2011; 108: 4516-4522.

Edgar RC. Search and clustering orders of magnitude faster than BLAST. Bioinformatics 2010; 26: 2460-2461.

Edgar RC, Flyvbjerg H. Error filtering, pair assembly and error correction for next-generation sequencing reads. Bioinformatics 2015; 31: 3476-3482.

Martin M. Cutadapt removes adapter sequences from high-throughput sequencing reads. EMBnet.journal; Vol 17, No 1: Next Generation Sequencing Data Analysis 2011.

Parada AE, Needham DM, Fuhrman JA. Every base matters: assessing small subunit rRNA primers for marine microbiomes with mock communities, time series and global field samples. Environ Microbiol 2016; 18: 1403-1414.

Rognes T, Flouri T, Nichols B, Quince C, Mahé F. VSEARCH: a versatile open source tool for metagenomics. Peerj 2016; 4.

Vesterinen EJ, Puisto AIE, Blomberg AS, Lilley TM. Table for five, please: Dietary partitioning in boreal bats. Ecol Evol 2018; 8: 10914-10937.

Vesterinen EJ, Ruokolainen L, Wahlberg N, Peña C, Roslin T, Laine VN, et al. What you need is what you eat? Prey selection by the bat *Myotis daubentonii*. Mol Ecol 2016; 25: 1581-1594.

Walters W, Hyde ER, Berg-Lyons D, Ackermann G, Humphrey G, Parada A, et al. Improved bacterial 16S rRNA Gene (V4 and V4-5) and fungal internal transcribed spacer marker gene primers for microbial community surveys. Msystems 2016; 1.
